# Widespread European outbreaks of *Salmonella* Enteritidis related to chicken meat from Poland, 2023

**DOI:** 10.1017/S0950268826102015

**Published:** 2026-09-24

**Authors:** Emily Dibba White, Maria João Cardoso, Alina Novacek, Felix Küffel, Norbert Handra, Christian Kornschober, Mihail Milanov, Gergana Mateva, Werner Ruppitsch, Anne Ribert Larsen, Malene Søby, Annemette Lyhne-Kjærbye, Stine Nielsen, Ruska Rimhanen-Finne, Anni Vainio, Aoife Colgan, Maria Pardos de la Grandara, Carolina Silva Nodari, Nathalie Jourdan Da Silva, Anika Meinen, Eva Trost, Eva Grilc, Marija Trkov, Rikard Dryselius, Ann Hoban, Marie Anne Chattaway, Anais Painset, Laura Villa, Claudia Lucarelli, Lin T. Brandal, Heidi Lange, Pernille Gymoese, Pernille Munch Ryder, Steen Ethelberg, Luise Müller, Sabine Maritschnik

**Affiliations:** 1 Statens Serum Institut, Denmark; 2 ECDC Fellowship Programme, (EPIET/EUPHEM), European Centre for Disease Prevention and Control, Sweden; 3 ECDC fellowship Programme, Public Health Microbiology path (EUPHEM), European Centre for Disease Prevention and Control, Sweden; 4 Austrian Agency for Health Food Safety, Austria; 5 National Diagnostic Research Veterinary Medical Institute Prof Dr G Pavlov, Bulgaria; 6 Medical University of Innsbruck Institute of Hygiene and Medical Microbiology, Austria; 7 Danish Veterinary and Food Administration, Denmark; 8 Finnish Institute for Health and Welfare, Finland; 9 Health Protection Surveillance Centre, Ireland; 10 Institute Pasteur, France; 11 Santé publique France, France; 12 Robert Koch Institute, Germany; 13 National Institute of Public Health of the Republic of Slovenia, Slovenia; 14 National Laboratory of Health Environment and Food Department for Public Health, Slovenia; 15 Public Health Agency of Sweden, Sweden; 16 UK Health Security Agency, UK; 17 Istituto Superiore di Sanità, Italy; 18 Norwegian Institute of Public Health, Norway; 19 University of Copenhagen Faculty of Health and Medical Sciences, Denmark

**Keywords:** *Salmonella*, disease outbreak, foodborne disease, genomics, intersectoral collaboration, chicken meat

## Abstract

*Salmonella* Enteritidis is typically transmitted to humans via consumption of eggs or poultry meat. In July 2023, Denmark and Austria concurrently reported two outbreaks of genetically distinct *S.* Enteritidis sequence type (ST) 11. In both countries, cases were identified as part of the outbreaks using core-genome Multilocus Sequence Typing (cgMLST) between 1^st^ of January and 1^st^ of October, 2023. Outbreak investigations were conducted and included interviews with cases, food tracing, and, using cgMLST, comparisons of human, food, and environmental isolates. In the EU, 207 cases across 11 countries were found. Patient food histories pointed towards chicken, often sold as kebab meat. Extensive traceback investigations from affected restaurants and wholesalers in Austria and Denmark identified chicken meat produced in Poland as the vehicle of the outbreaks. Due to the death of one patient in Austria, the food safety authorities initiated more food safety controls for this food product, which led to food recalls. Recalls of specific chicken products from Poland were also conducted in Denmark. Despite national interventions and regulations targeting poultry producers, the EU remains collectively vulnerable to large-scale food contamination events originating in a single member state. Effective international and cross-sector collaboration was crucial to identifying the vehicle of these outbreaks.

## Introduction

Salmonellosis is a major cause of bacterial enteric illness in Europe and is characterized by diarrhoea, fever, abdominal pain and/or vomiting [1]. It is primarily transmitted through contaminated and undercooked food items, including eggs, poultry and meat products, and green produce [2]. The incidence of salmonellosis in 2023 in the EU/EEA was 18.1 cases per 100,000 population, with *S.* Enteritidis emerging as the most frequently reported serovar [3].

*S.* Enteritidis exhibits the capability to infect the ovaries of chickens, subsequently posing a risk of infecting humans through the ingestion of contaminated poultry meat and eggs. Its entry into industrialized broiler and egg production in the Western World led to an unprecedented pandemic, resulting in millions of human infections, particularly during the 1980s and 1990s. However, targeted interventions implemented at both national and EU levels have taken steps towards controlling salmonellosis within the EU [4], although it still causes significant foodborne illness in Europe. Denmark represents an unusual case in this regard; *S.* Enteritidis occurs only sporadically in commercially raised chicken broilers and layers since 2013, and chicken meat undergoes strict testing for *Salmonella* contamination before market release [5, 6]. As a result, human cases are comparatively few and predominantly travel-associated, with domestic outbreaks being rare. In Austria, *S.* Enteritidis has also been controlled through implementation of a national control system, and human cases have been gradually reduced over the past decades [7].

In July 2023, Denmark reported a cluster of *S.* Enteritidis ST11 cases identified by whole-genome sequencing (WGS) through the ECDC EpiPulse portal (*Outbreak-1*). Several EU Member States (MSs), including Austria, reported patients with matching strains and a cross-border investigation was initiated. Concurrently, an investigation of another distinct *S.* Enteritidis ST11 outbreak (*Outbreak-2*) was initiated by Austria, following a fatal case at the beginning of July 2023. Outbreak investigations of *S.* Enteritidis cases with isolates belonging to both subtypes were conducted by the Austrian Agency for Health and Food Safety (AGES) and, in Denmark, by the Statens Serum Institut (SSI). The European Centre for Disease Prevention and Control (ECDC) gathered outbreak data from additional EU MSs. The evidence pointed towards poultry meat produced in Poland. In October 2023, ECDC published a joint Rapid Outbreak Assessment together with the European Food Safety Authority (EFSA) summarising the information available at the time [8].

This paper presents outbreak data across Europe and describes the microbiological, epidemiological, and food investigations undertaken in Austria and Denmark during these two concurrent cross-border *S.* Enteritidis outbreaks.

## Methods

### Case definitions

For Outbreak-1, a case was a person with laboratory-confirmed *S.* Enteritidis ST11 complex type (CT)2,114 (equivalent to HC2_352197 and HC2_61234 using Enterobase’s hierarchical clustering scheme) with disease onset from 1 January to 1 October 2023 (date of sampling or date of receipt by the laboratory if date of onset is not available), and within five allelic differences (AD) from the Danish reference strain, using the cgMLST scheme comprising 3,002 target genes for *S. enterica* 9. For Outbreak-2, a case was a person with laboratory-confirmed *S.* Enteritidis ST11 CT13755 (equivalent to HC5_283929 using Enterobase’s hierarchical clustering scheme) with disease onset from 1 January to 1 October 2023 (date of sampling or date of receipt by the laboratory if date of onset is not available), and within five allelic differences (AD) from the Austrian reference strain, using the cgMLST scheme comprising 3,002 target genes for *S. enterica* 9. Deaths were recorded as non-vital status within 30 days after the sample date. The case definitions were shared with the other EU MS through EpiPulse together with a joint linelist template, for the MS countries to fill in. The linelist included information about sex, age, sampling date, hospitalization, vital status, travel abroad, and consumption of chicken and kebab within 7 days prior to illness for each case. Exposure information was compiled from all participating countries and included in the overall analysis whenever such data were available.

### Human investigations

In Denmark, as part of routine surveillance, *Salmonella* isolates from positive samples collected from infected persons were sent to SSI and whole-genome sequenced in-house. The investigation by the SSI involved telephone interviews with confirmed cases, using hypothesis-generating questionnaires (Supplementary Material 2) covering demographics, clinical onset, symptoms, and food consumption seven days prior to disease onset. The questionnaire covered various food items and their sources. Initial interviews covered all items, but suspicion towards chicken and kebab led to a revised, shorter questionnaire focusing on these, in situations where consumption of chicken or kebab was reported. Cases were interviewed promptly upon reporting.

In Austria, as part of surveillance, *Salmonella* isolates were routinely whole-genome sequenced in-house at the National Reference Centre for *Salmonella* at AGES (NRL-AGES). *Salmonella* isolates from food specimens were likewise collected and whole-genome sequenced at NRL-AGES routinely.

A descriptive epidemiological investigation of the outbreak was coordinated and conducted by AGES. A standard hypothesis-generating questionnaire (Supplementary Material 3) for *Salmonella* was used for telephone interviews of cases. Cases were inquired about demographics, clinical onset, symptoms, and their purchasing behaviour and eating habits prior to illness. Active case finding was conducted through interviewing confirmed cases about further people known to have fallen ill with diarrhoea.

### Food investigations

In Denmark, isolates from food products under suspicion of being outbreak-related were whole-genome sequenced by The Danish Veterinary and Food Administration (DVFA). Based on patient interviews, supplier information was obtained for chicken kebabs and other chicken meat products in 13 restaurants. Traceback investigation of consumed chicken products was performed by the DVFA to determine the origins of the products. Traceforward was carried out in order to uncover the distribution of chicken products in Denmark and confirm the suspicion of the source of Outbreak-1. Food products were collected at the importer, wholesalers and retailers, examined for *Salmonella* and, if positive, compared to human cases by WGS, using cgMLST (see Supplementary Material). On 25 August 2023, DVFA created an alert notification in the Rapid Alert System for Food and Feed (RASFF; ref. no 2023.5788), describing the outbreak. Moreover, the authorities in Poland were asked to follow up on the notification and share the results of their investigations.

In Austria, the provincial food safety authorities conducted investigations to explore the supply chains of food products and food service establishments identified during case interviews. The epidemiological outbreak investigation involved interviewing confirmed cases to identify potential food vehicles. These interviews showed common patterns of food consumption at specific restaurants, prompting the food safety authority to collect samples from these establishments and initiate traceback investigations. The restaurants were selected based on exposure reported during case interviews in relation to the date of visit and date of disease onset. For outbreak-1, 3 food service establishments were investigated with traceback, and for outbreak-2, 8 food service establishments were investigated with traceback. Additionally, further investigations were carried out at food service establishments serving kebabs to assess food safety. Information necessary for food traceback processes was provided by food retailers, distributors, importers, and representative public health agencies from various countries. The detection of *Salmonella* spp. in food and environmental samples from food service establishments prompted the initiation of RASFF reports. This ensured timely notification to other member states about the removal of contaminated products from the market. The RASFF reports (RASFF numbers presented in Table 1) provided detailed information for the food supply chain that was associated with the contaminated food identified.

**Table 1.** Comparison of *Salmonella* Enteritidis ST11 Outbreak-1 and Outbreak-2 in Denmark and Austria, countries with interviews of the cases and traceback investigations, 1 January–1 October 2023 Table 1. long description.

	Outbreak-1		Outbreak-2	
Complex type	CT 2114		CT13755	
EpiPulse event	2023-FWD-00045		2023-FWD-00048	
Total number of cases in ROA	(*N* = 192)		(*N* = 66)	
Country	Denmark	Austria	Denmark	Austria
Number of cases	28	6	1	11 (1 fatal)
Age range	9–98	7–75	–	1–63
Median age	29.0	13.5	–	21
Hospitalisations	8	1	Unknown	4
Month of occurrence (2023)	May–September	March–June	September	April–August
Number of cases interviewed	24	5	1	11
# Provinces affected	5	6	1	4
RASFF messages	RASFF 2023.5788	RASFF 2023.5055	–	RASFF 2023.5055 RASFF 2023.6079 RASFF 2023.4360 RASFF 2023.6418
Number of cases that consumed chicken/kebab/pizza	17/24	5/6	Unknown	11/11
Matching food and environmental isolates	1 Frozen chicken product	1 Chicken skewer (MRS-23/01527); 3 Cleaning cloth (MRS-23-01536, MRS-23-01544, MRS-23-01551)	0	3 Chicken skewers (MRS-23/01528, MRS-23/01529, MRS-23/01530); 1 Cleaning cloth (MRS-23/01718)
Sampling week (2023) of food and environmental isolates	Week 33	Week 28	–	Week 28

## Results

**Figure 1.**
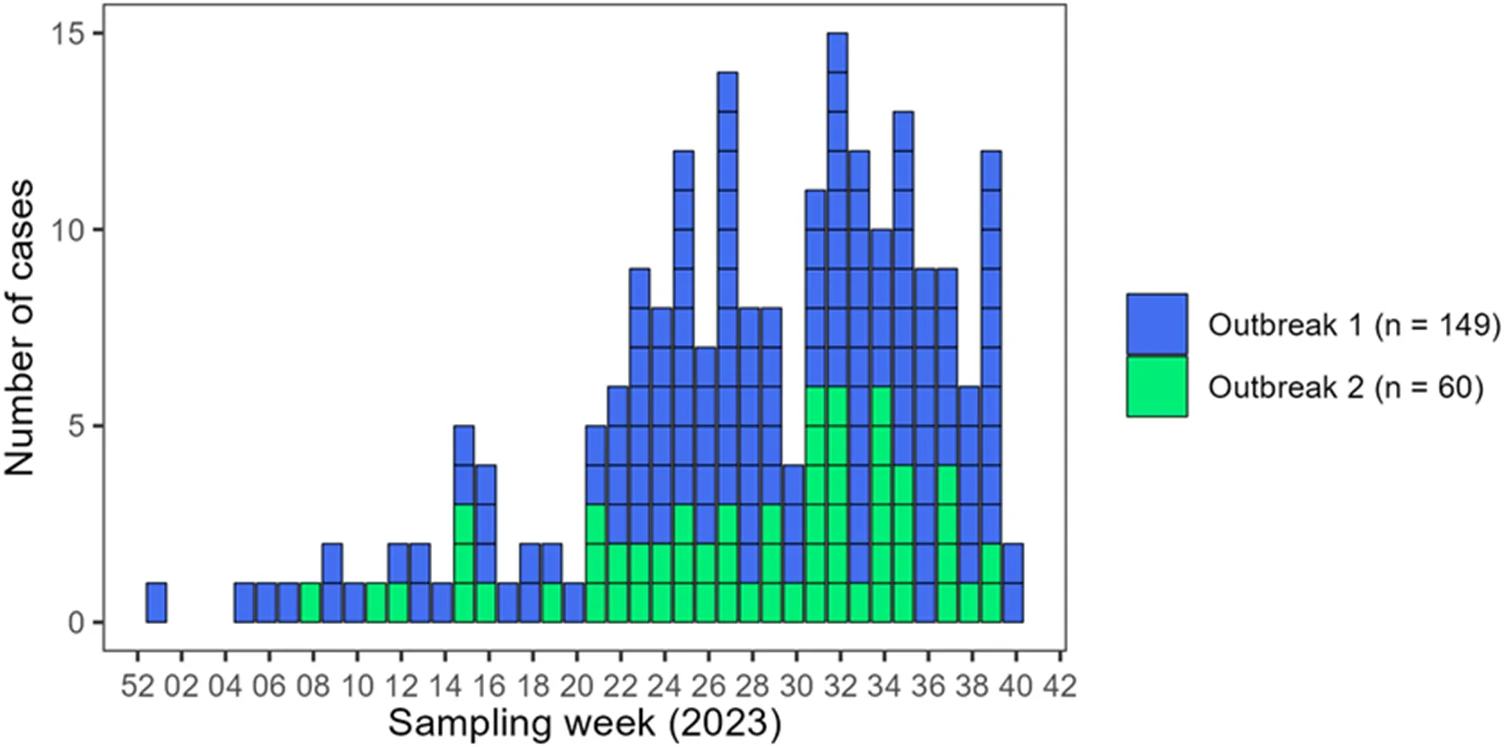
Number of *Salmonella* Enteritidis ST11 cases in Outbreak-1 and Outbreak-2, from all countries with reported cases per week of sample (n=209), 1 January – 1 October 2023.

Table 1 presents a comparative overview of cases from Denmark and Austria for outbreak-1 and outbreak-2. In Denmark, 28 cases fulfilled the case definition for Outbreak-1, while one case met the case definition for Outbreak-2. Cases appeared almost every week from May to September 2023. The median age of the patients was 29.5 years (range 9–98 years), and 57% were males. Cases lived in all five of the Danish administrative regions, indicating a widely distributed outbreak vehicle. The hospitalisation proportion was 29%, and no deaths were recorded. In Austria, six and 11 cases fulfilled the case definition for Outbreak-1 and Outbreak-2, respectively. For Outbreak-1, the disease onset was from March to June 2023. The median age was 13.5 years (range 7–75 years). All cases were male and originated from six different (out of eight) provinces. For Outbreak-2, the median age was 21 years (range 1–63 years), 72% of cases were male, they lived in four different provinces, and did not report travel history. A total of 36% were hospitalized, and one case was fatal. Figure 1 presents the number of cases by time for both outbreaks, showing the numbers of reported cases over time from all countries that have reported cases. Table 2 details demographic information on the cases from all countries that reported cases.

**Table 2.** Number *of Salmonella* Enteritidis ST11 cases, hospitalisation, travel history, consumption of chicken and kebab in Outbreak-1 and Outbreak-2, from countries with reported cases, 1 January–1 October 2023 Table 2. long description.

	Outbreak-1 (*N* = 149)	Outbreak-2 (*N* = 60)	Total (*N* = 209)
Sex			
Female	66 (44.3%)	18 (30.0%)	84 (40.2%)
Male	81 (54.4%)	41 (68.3%)	122 (58.4%)
Unknown	2 (1.34%)	1 (1.67%)	3 (1.44%)
Age group (years)			
0–10	8 (5.4%)	3 (5.0%)	11 (5.3%)
11–20	26 (17.4%)	14 (23.3%)	40 (19.1%)
21–30	31 (20.8%)	16 (26.7%)	47 (22.5%)
31–40	20 (13.4%)	5 (8.3%)	25 (12.0%)
41–50	15 (10.1%)	4 (6.7%)	19 (9.1%)
51–60	14 (9.4%)	4 (6.7%)	18 (8.6%)
61–70	20 (13.4%)	3 (5.0%)	23 (11.0%)
71–80	8 (5.4%)	1 (1.7%)	9 (4.3%)
81+	7 (4.7%)	10 (16.7%)	17 (8.1%)
Unknown	0 (0.00%)	0 (0.00%)	0 (0.00%)
Hospitalized			
Yes	13 (8.7%)	10 (16.7%)	23 (11.0%)
No	24 (16.1%)	10 (16.7%)	34 (16.3%)
Unknown	112 (75.2%)	40 (66.7%)	152 (72.7%)
Travel abroad			
Yes	21 (14.1%)	5 (8.3%)	26 (12.4%)
No	49 (32.9%)	25 (41.7%)	74 (35.4%)
Unknown	79 (53.0%)	30 (50.0%)	109 (52.2%)
Country			
Austria	6 (4.0%)	11 (18.3%)	17 (8.1%)
Denmark	28 (18.8%)	1 (1.7%)	29 (13.9%)
Finland	3 (2.0%)	1 (1.7%)	4 (1.9%)
France	35 (23.5%)	14 (23.3%)	49 (23.4%)
Germany	3 (2.0%)	11 (18.3%)	14 (6.7%)
Ireland	1 (0.7%)	0 (0%)	1 (0.5%)
Italy	2 (1.3%)	1 (1.7%)	3 (1.4%)
Norway	0 (0%)	4 (6.7%)	4 (1.9%)
Slovenia	0 (0%)	2 (3.3%)	2 (1.0%)
Sweden	2 (1.3%)	4 (6.7%)	6 (2.9%)
United Kingdom	69 (46.3%)	11 (18.3%)	80 (38.3%)
Chicken consumption			
Yes	25 (16.8%)	16 (26.7%)	41 (19.6%)
No	0 (0%)	1 (1.7%)	1 (0.5%)
Unknown	124 (83.2%)	43 (71.7%)	167 (79.9%)
Kebab consumption			
Yes	24 (16.1%)	11 (18.3%)	35 (16.7%)
No	5 (3.4%)	9 (15.0%)	14 (6.7%)
Unknown	120 (80.5%)	40 (66.7%)	160 (76.6%)

In Denmark, when 12 cases (Outbreak-1) had been reported, an alert was sent via the ECDC EpiPulse communication channel (2023-FWD-00045; 21 July 2023) to inform other countries in the EU about the outbreak and share the outbreak strain sequence information. Overall, 24 of 28 cases were interviewed. Early responses quickly pointed to kebab meat as the suspected vehicle.

**Figure 2.**
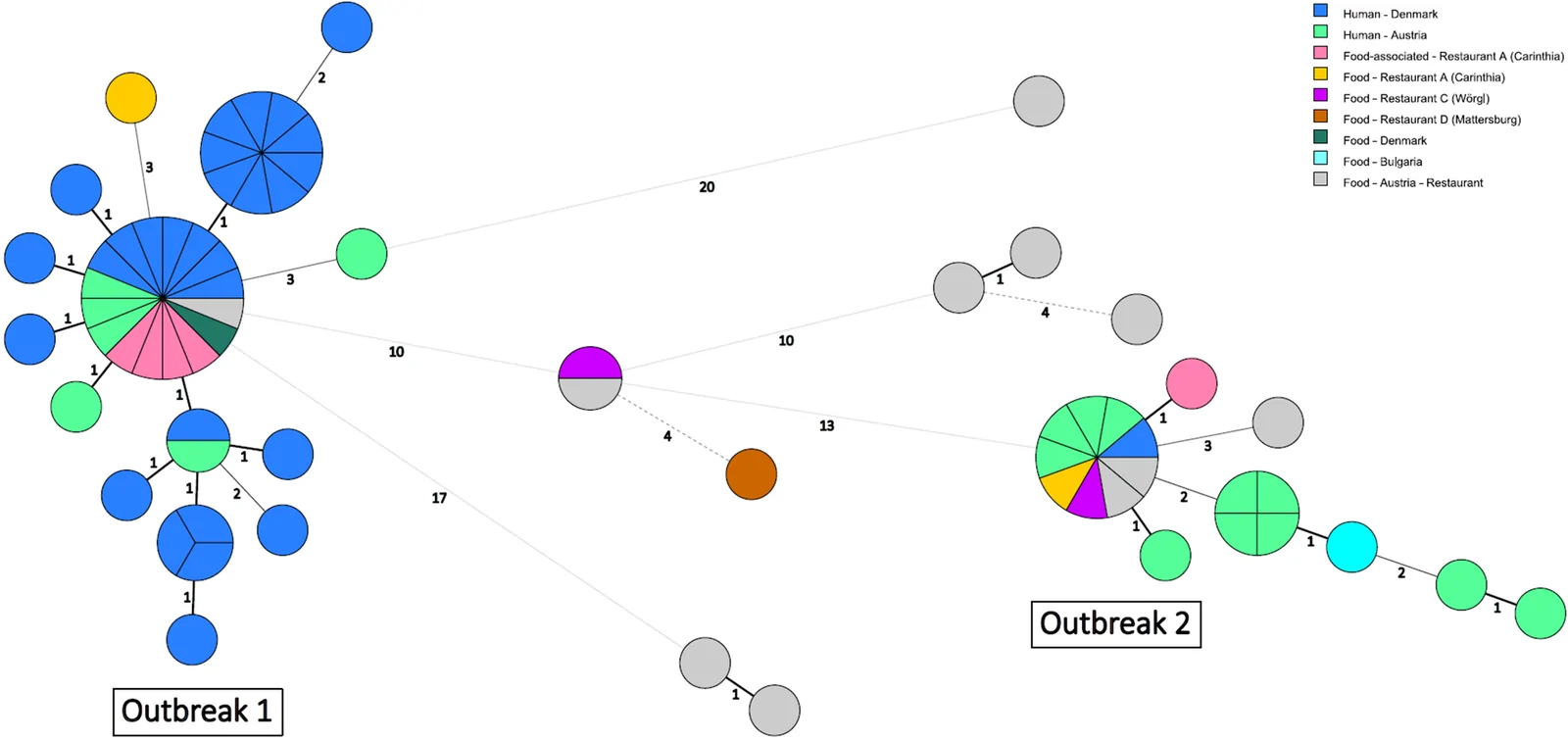
Minimum spanning tree of 69 isolates of *Salmonella* Enteritidis ST11 in two outbreaks, 2023.

In Austria, all Outbreak-1 cases reported eating chicken and/or kebab meat. Five out of six had been eating at restaurants prior to becoming ill. For Outbreak-2, case interviews showed that all eleven cases had consumed chicken prior to illness. Among them, five reported having consumed chicken kebab in various restaurants (Table 2). Comparative cgMLST analysis of isolates submitted through the EpiPulse platform revealed that the Danish and Austrian outbreak strains were closely related and both detected in one sealed, frozen, and raw chicken kebab specimen as well as a cleaning cloth from the same restaurant (Restaurant A) (Figure 2). This connection indicated a common vehicle contaminated with two genetically distinct outbreak strains. Outbreak strains were closely related; however, only the strain from Outbreak-2 was isolated from the fatal case who dined at Restaurant A. Moreover, the cgMLST data linked additional cases from other countries to these clusters, suggesting international distribution of the implicated product.

### Food investigations

**Figure 3.**
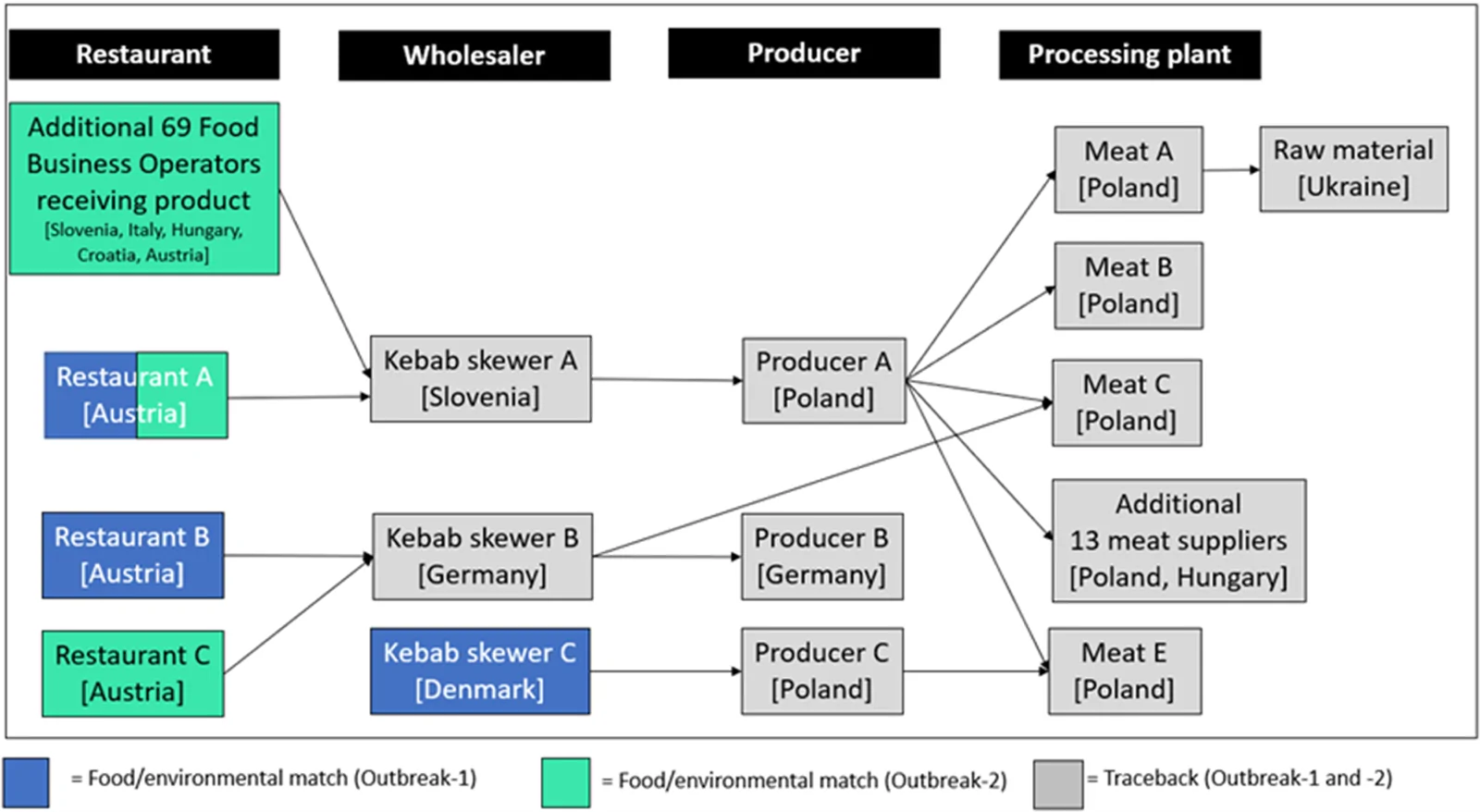
Traceback of the meat and meat products from production to sale.

In July 2023, the Public Health Directorate in the Austrian province of Carinthia initiated an extensive food inspection and traceback investigation, starting at the restaurant [Restaurant A] where the fatal case had eaten before falling ill. *S.* Enteritidis ST11 strains matching Outbreak-1 and Outbreak-2 were found in a food-associated isolate (microfiber cleaning cloth) and food (sealed, frozen, raw chicken kebab), respectively. Figure 2 presents the isolates from the Danish and Austrian human cases, food isolates from Restaurant A, C, and D in Austria, a food-associated isolate from Restaurant A in Austria, and other food isolates from Denmark, Austria, and Bulgaria. Figure 3 shows interconnected traceback investigations where consumption of chicken had been reported by cases at specific food business operators (FBOs); these show connections in the supply chain between both countries and a common country of origin for the chicken meat. Traceback of the frozen and raw chicken kebab revealed a Slovenian distributor, but sourced from Poland [Producer A] (Figure 3). The meat for the kebab skewer originated from three different processing plants, two located in Poland [Meat B + C] and one in Ukraine [Meat A]. Furthermore, traceback investigation of the chicken kebab consumed by other cases in Outbreak-2 revealed a German wholesaler, who had obtained the chicken kebab skewer from one of the previously mentioned processing plants in Poland [Meat C]. In addition, traceback investigations of Outbreak-1 could be linked to the same German wholesaler mentioned above, highlighting a connection in the supply chain distribution network (Figure 3).

On 4 August 2023, an Early Warning and Response System (EWRS) notification was issued, along with a “selective exchange” with Poland. This notification conveyed that three isolates from the frozen, raw chicken kebab skewer sampled at Restaurant A matched the Outbreak-2 strain (with 0–1 allelic differences, Figure 2). Additionally, one isolate from the same frozen, raw chicken kebab skewer and 22 other isolates from the microfiber cleaning cloth displayed 2 or 0–1 allelic differences to the Outbreak-1 strain, respectively.

In September 2023, the DVFA found *S.* Enteritidis ST11 matching the Outbreak-1 strain by WGS in imported pre-cooked frozen chicken kebab [Kebab skewer C]. The product was collected from a wholesaler with sales to restaurants. The recall was based on the positive test. The contaminated batch was investigated due to interview results and traceback and recalled from the market by the Danish importer; however, based on case information, further batches were likely also contaminated. DVFA found that the common kebab producer was [Producer C] from Poland.

In September 2023, a food investigation was additionally conducted by the food safety authority in Bulgaria. *S.* Enteritidis ST11 isolates were obtained during an official inspection at a local wholesaler in a batch of frozen chicken wings. These isolates were shared with AGES for sequencing, revealing a match to Outbreak-2 and reported in a RASFF notification (ref no 2023.4360). In addition, isolates of *S.* Enteritidis ST11 CT1483, *S. infantis*, and *S.* Kottbus were found. The chicken wing batches had been produced in Poland [Producer B], which was already previously known to be associated with Outbreak-2 (Figure 3).

On 4 September 2023, Polish authorities informed via RASFF that the raw meat used in chicken kebabs was likely the source of the *Salmonella* contamination. Raw, marinated chicken meat was used to prepare the skewers, which were subsequently frozen and distributed. In Austria, AGES identified challenges in maintaining the required cold chain conditions, specifically keeping the frozen, raw chicken skewers below 0°C throughout the distribution process before being cooked at the various food service establishments. Additionally, they reported that the cut meat had been roasted at a temperature of 80–85°C, however, with a risk of undercooked pieces of meat present in the packages. Undercooked pieces of meat could become detached from the body of meat during searing and may not reach the desired temperature. Corrective and preventive measures were implemented by the authorities in Poland according to the rapid outbreak assessment [8].

In summary, out of 27 samples taken in Austria and Denmark from chicken kebab between July and November 2023, 16 tested positive for *Salmonella* spp. and were reported in 5 RASFF reports. However, 11 positive samples could not be included in the European system due to a lack of evidence (missing invoices and delivery documents). Five reports identified new food business operators linked to contaminated poultry from Poland, while two reports uncovered additional connections to previously identified traceback chains associated with Outbreak-1 and Outbreak-2. These food traceback investigations uncovered numerous connections, highlighting a complex interdependence among the three interconnected supply chains. WGS thus established connections between food business operators and, further, additional *S.* Enteritidis ST11 isolates and other serovars with different complex types from various kebab and chicken meat samples with an origin in Poland were identified. These were found to be closely related from a genomic point of view, despite not corresponding to either of the outbreaks’ strains (Figure 2).

### Outbreak cases across Europe

The international communication, as coordinated by the ECDC, led to the identification of cases with isolates matching the outbreak strains being reported from a number of countries beyond Denmark and Austria [8]. Thus, between 1 January and 1 October 2023, a total of 209 laboratory-confirmed *S.* Enteritidis ST11 cases of Outbreak strain 1 or 2 were reported by 11 EU MS countries and the United Kingdom (UK) (Table 2). The demographic characteristics were similar to those seen in Denmark/Austria; overall, the median age of cases was 28 years (range 1–98 years), and two-thirds were male. Among cases with available information, 23 were hospitalized, and one died, within 30 days from sample-date. Travel abroad prior to disease onset was reported for 26 cases, but to different locations. Of the 7 out of 12 countries for which food consumption information was known, 41/42 cases and 35/49 cases had consumed chicken and/or kebab, respectively.

## Discussion

We describe outbreaks with two concurrent and distinct *S.* Enteritidis ST11 strains affecting a number of European countries in 2023. The source was traced to poultry meat produced in Poland, distributed and sold in all affected countries. This source was identified through national outbreak investigations, conducted collaboratively mainly in two EU MSs (Denmark and Austria) where 46 confirmed cases occurred. Close collaboration between Denmark and Austria was established early in the investigation through the EpiPulse platform, where epidemiological and microbiological information was continuously exchanged. Regular communication between the national focal points and food safety authorities enabled rapid alignment of case definitions, comparison of genomic data, and coordination of traceback activities. The outbreak investigation followed a stepwise approach, integrating epidemiological interviews, whole-genome sequencing, and food chain analyses to identify the likely source. Comparative cgMLST analysis revealed two closely related *S.* Enteritidis clusters linked to the same food establishment, prompting coordinated traceback through national control authorities, which ultimately identified a common supplier. Following identification of the contaminated product, both countries implemented control measures, including product withdrawal, public communication, and enhanced surveillance for new cases. The collaboration facilitated timely exchange of findings across sectors and countries, illustrating the added value of cross-border coordination for managing foodborne outbreaks.

Further comparison of WGS information across Europe identified an additional 209 outbreak cases in 9 additional EU countries and the UK, from 1 January to 1 October 2023. It is important to note that the reported case numbers represent only those patients diagnosed with *Salmonella*, captured by surveillance and where isolates were successfully sequenced. This will represent only a fraction of the actual number of cases occurring, in particular as surveillance and WGS coverage vary widely across countries in Europe [9]. Consequently, the true number of people affected by these prolonged outbreaks may have been substantial, in fact representing a major public health problem. These outbreaks have previously been published in a Rapid Outbreak Assessment report by ECDC and EFSA [8]. While the ECDC–EFSA report provided a Europe-wide overview of this outbreak, our study offers complementary insights into the operational aspects of the joint investigation. Specifically, we describe the country-level collaboration, decision-making processes, and coordination mechanisms that underpinned the successful identification and control of the outbreak source. These details highlight how structured information-sharing and multidisciplinary coordination can strengthen outbreak response capacity in a cross-border context.

An important element in the outbreaks we describe was that many cases were infected at restaurants or fast-food establishments. Descriptive epidemiology, involving detailed food histories, allowed us to identify a series of food vendors where the transmission had likely taken place. Many of the food vendors served chicken or kebab meat, and traceback through the food vendors supported identification of the suppliers. Several food retailers with positive food specimens for *Salmonella* spp. showed multiple strains within the contaminated chicken meat specimens tested. All implicated batches of frozen chicken and mixed kebab meat were produced in Poland using Polish and Ukrainian meat and distributed to several EU countries, resulting in widespread contamination and outbreaks. Traceback and traceforward investigations conducted in both Austria and Denmark linked these food establishments to different slaughterhouses and meat processing companies in the same country. These findings were corroborated by the fact that traceback efforts by both Austrian and Danish food authorities pointed to the same suppliers. As a result, analytical epidemiological studies were not conducted.

Numerous previous outbreak investigations have shown the value of applying WGS to bacterial isolates [10–15]. For this outbreak, we applied WGS for the outbreak case-definition, which enabled the identification of cases in both Austria and Denmark as part of the same outbreak. Furthermore, WGS facilitated case finding in a series of other European countries that all used WGS, either partially or fully, as part of their national *Salmonella* surveillance. This was possible through the assistance of ECDC, which coordinated the sequence comparisons and facilitated the communication about sequence types through their designated outbreak communication platform EpiPulse. Beyond defining the outbreak, WGS also assisted in solving the outbreak by allowing comparisons between patient isolates and with isolates from food products or specimens from food vendor environments. Communication about findings in foods and official inquiries made to the Polish food authorities were facilitated via the EU’s communication system for risk associated with foods, RASFF. Thus, the strong discriminatory power of WGS coupled with efficient transnational communication channels was an important reason for the success of this international outbreak investigation. International cooperation and information sharing are crucial for the timely detection, investigation, and containment of outbreaks. This has also been exemplified in a recently published multi-country outbreak of *S.* Mbandaka linked to pre-cooked, frozen chicken meat in ready-to-eat products in Finland, where coordinated epidemiological investigations, genomic surveillance, and cross-border collaboration enabled identification and source tracing of products produced outside of the EU [16]. Collaboration among government agencies, industry stakeholders, and scientific communities can facilitate the development and implementation of harmonized standards and best practices aimed at reducing the prevalence of *Salmonella* contamination in poultry products.

Several factors may have contributed to the risk of S*almonella* contamination in poultry production and processing [17]. These include inadequate hygiene practices, suboptimal biosecurity measures, improper storage and transportation conditions, and gaps in regulatory oversight. Additionally, certain management practices in intensive poultry production systems, such as high animal density, may facilitate pathogen transmission among flocks and increase the risk of contamination. However, the present investigation did not identify which specific factors within the implicated production companies may have contributed. Effective control and mitigation strategies are essential for preventing and managing *Salmonella* outbreaks associated with poultry products [18]. Intensified food inspections by the Austrian food safety authorities among chicken kebab meat food retailers between August and September 2023 revealed positive food specimens and new connections among food retailers and food business operators. Stringent controls (e.g., frequent hygiene inspections and microbiological testing of food sampling of products served) of retailers serving chicken kebab in Austria are ongoing.

Comprehensive risk-based approaches encompassing the entire food production continuum, from farm-to-fork, are imperative. These measures may include the implementation of Hazard Analysis and Critical Control Points (HACCP) systems, stringent hygiene protocols, regular monitoring and testing of poultry flocks and processing facilities, as well as targeted interventions to address specific risk factors identified in the production chain. Since *Salmonella* is not killed during the freezing process, cross-contamination poses a significant risk for food business operators [19, 20]. This risk is especially heightened during periods of high demand at food business operators, where the pace of food preparation may compromise safety standards. In such scenarios, meat may be cut and handled too quickly, leading to inadequate cooking temperatures that do not effectively kill pathogens like *Salmonella.* It is presumed that improper handling practices, including inadequate temperature control during storage and insufficient heating prior to consumption, contributed to the risk of bacterial growth. In other words, it seems possible that the rotating frozen, raw chicken kebab skewer may not always reach adequate temperatures and therefore kebab meat could play a crucial role in the spread of *Salmonella* in a busy restaurant.

Similar large-scale outbreaks of *S.* Enteritidis have also been linked to eggs and egg products in Europe. Eggs remain a well-recognized vehicle for *S.* Enteritidis infection, including several outbreaks associated with eggs from Poland. Between 2015 and 2019, a total of 612 foodborne outbreaks (340 strong-evidence and 272 weak evidence) due to *S.* Enteritidis in eggs and egg products were reported to EFSA by 19 EU Member States, involving 4,481 cases and 1,087 hospitalisations, and 10 deaths [21]. In 2020, 48 further outbreaks linked to eggs and egg products were reported to EFSA by seven EU countries, and international outbreaks occurred in 2021–2022. The largest European multi-national outbreak between 2015 and 2018 involved 838 WGS-confirmed and 371 probable cases in 18 EU and EEA countries and was traced to eggs from Poland [22]. Control measures implemented in 2016 initially reduced case numbers, but a resurgence occurred in 2017–2018 [23]. In regard to policies and practices regarding *Salmonella* surveillance, the EU MSs may be very different. Denmark has a long history of controlling *Salmonella* in the broiler and table egg production. The first Danish *Salmonella* control programme in the production of broilers and table eggs came into effect on 1 January 1994. It was approved by the European Commission as an implementation of the Council Directive 92/117/EEC. Since then, Denmark has implemented extended surveillance and control programmes in the table egg and broiler production, which include regular and frequent sampling and testing of flocks for *Salmonella*. As a result of these programmes, Denmark has a very low prevalence of *Salmonella* in poultry (*Gallus gallus*). Furthermore, in 2007, Denmark applied for special guarantees for *Salmonella* in table eggs and broiler meat, and these were granted by the European Commission in 2012 and 2018, respectively. The very low occurrence of *Salmonella* in table eggs and broiler meat is maintained through the extensive surveillance and control programmes throughout the production systems, which aim to detect infected flocks as early as possible and prevent *Salmonella* from reaching the consumer. For example, eggs from flocks suspected of being infected with *Salmonella* are all sent for heat treatment/destroyed. This could indeed contribute to the different levels of *Salmonella* between Polish and Danish eggs and chicken. *S.* Enteritidis outbreaks linked to imported poultry products highlight the importance of maintaining strong food safety controls across borders, as differences in production practices and standards may have implications for both public health and international trade [24]. The occurrence of such outbreaks may lead to trade disruptions, import bans, and economic losses for affected countries and stakeholders. Consequently, there is a growing relevance for transparent and science-based risk assessment frameworks to inform trade policies and regulations to maintain consistent food safety among globally traded food products.

## Conclusion

In conclusion, this investigation shows the power of using international food traceback as an instrument for solving outbreaks. It also emphasizes the importance of widespread implementation of whole-genome sequencing and the ability to share and compare sequencing data on human and food isolates across countries. Utilising a common platform such as EpiPulse enhances collaboration and strengthens the collective response to public health threats. The emergence of *S.* Enteritidis outbreaks linked to Polish chicken underscores the complex challenges and multifaceted nature of foodborne illness prevention and control in an increasingly interconnected world. Addressing these challenges requires a unified effort from all stakeholders, including governments, regulatory agencies, industry partners, and consumers, to implement comprehensive and evidence-based strategies aimed at mitigating the risk of *Salmonella* contamination in poultry products and safeguarding public health on a global scale.

## Supporting information

10.1017/S0950268826102015.sm001 White et al. supplementary material White et al. supplementary material

## Data Availability

Sequences are available through SRA as bioproject PRJNA1243087 and PRJEB91269.

## Supplementary material

The supplementary material for this article can be found at http://doi.org/10.1017/S0950268826102015.

## Long descriptions

### [Table 1] Long description

The table compares two outbreaks across several epidemiological and investigative parameters.

Outbreak-1 (Complex type C T 2114, EpiPulse 2023-F W D-00045, N equals 192):

* Denmark: 28 cases, age range 9 to 98 (median 29.0), 8 hospitalizations, occurring May to September. 24 cases interviewed, 5 provinces affected. R A S F F 2023.5788. 17 of 24 cases consumed chicken/kebab/pizza. 1 frozen chicken product isolate from week 33.

* Austria: 6 cases, age range 7 to 75 (median 13.5), 1 hospitalization, occurring March to June. 5 cases interviewed, 6 provinces affected. R A S F F 2023.5055. 5 of 6 cases consumed chicken/kebab/pizza. Isolates include 1 chicken skewer and 3 cleaning cloths from week 28.

Outbreak-2 (Complex type C T 13755, EpiPulse 2023-F W D-00048, N equals 66):

* Denmark: 1 case in September, age and median age not provided. Hospitalization status unknown. 1 case interviewed in 1 province. No R A S F F or matching isolates reported.

* Austria: 11 cases (1 fatal), age range 1 to 63 (median 21), 4 hospitalizations, occurring April to August. 11 cases interviewed, 4 provinces affected. Multiple R A S F F messages (2023.5055, 2023.6079, 2023.4360, 2023.6418). 11 of 11 cases consumed chicken/kebab/pizza. Isolates include 3 chicken skewers and 1 cleaning cloth from week 28.

Navigate back to [Table 1].

### Table 2 Long description

The table presents data for Outbreak-1 (N = 149), Outbreak-2 (N = 60), and a Total (N = 209).

* Sex: Males represent the majority in both outbreaks (54.4% in Outbreak-1, 68.3% in Outbreak-2, 58.4% total).

* Age group: The most affected groups are 11 to 20 years (19.1% total) and 21 to 30 years (22.5% total). Outbreak-2 shows a notable concentration in the 81 plus age group (16.7%).

* Hospitalized: 11.0% of total cases were hospitalized, though data is unknown for 72.7% of cases.

* Travel abroad: 12.4% of total cases reported travel, while 52.2% are unknown.

* Country: The United Kingdom reported the highest number of cases (80 cases, 38.3%), followed by France (49 cases, 23.4%) and Denmark (29 cases, 13.9%). Outbreak-1 was heavily concentrated in the United Kingdom (46.3%), while Outbreak-2 was more evenly distributed between France (23.3%), Austria (18.3%), and Germany (18.3%).

* Chicken consumption: 19.6% of total cases confirmed consumption, with 79.9% unknown.

* Kebab consumption: 16.7% of total cases confirmed consumption, with 76.6% unknown.

Navigate back to Table 2.
