# Supplementary material for: Widespread European outbreaks of Salmonella Enteritidis related to chicken meat from Poland, 2023
Source: Epidemiol Infect. 2026 Sep 24;154:e118. doi: 10.1017/S0950268826102015 (PMC13749103; doi:10.1017/S0950268826102015)
Supplement: Supplementary file 1 [file S0950268826102015sup001.zip › 3. Salmonella_enteritidis_Supplementary_final_version.docx]

**Supplementary Material**

Widespread European outbreaks of *Salmonella* Enteritidis related to chicken meat from Poland, 2023

Emily Dibba White^1,2^, Maria João Cardoso^3,19^, Alina Novacek^3^, Felix Küffel ^3^, Norbert Handra^3^, Christian Kornschober^3^, Mihail Milanov^4^, Gergana Mateva ^4^, Werner Ruppitsch^18^, Anne Ribert Larsen^5^, Malene Søby^5^, Annemette Lyhne-Kjærbye^5^, Stine Nielsen^1^, Ruska Rimhanen-Finne^7^, Anni Vainio^7^, Aoife Colgan^8^, Maria Pardos de la Gandara^9^, Carolina Silva Nodari^9^, Nathalie Jourdan-da Silva^10^, Anika Meinen^11^, Eva Trost^11^; Eva Grilc^12^, Marija Trkov^13^, Rikard Dryselius^14^, Annie Hoban^15^, Marie Chattaway^15^, Anais Painset^15^, Laura Villa^16^, Claudia Lucarelli^16^, Lin T. Brandal^17^, Heidi Lange^17^, Pernille Gymoese^1^, Pernille Kold Munch^1^, Steen Ethelberg^1,6^, Luise Müller^1^, and Sabine Maritschnik^3^

*^1^ Statens Serum Institut, Copenhagen, Denmark*

*^2^* *ECDC Fellowship Programme, (EPIET/EUPHEM), European Centre for Disease Prevention and Control (ECDC), Stockholm, Sweden*

*^3^ Austrian Agency for Health and Food Safety, Vienna, Austria*

*^4^ National Diagnostic and Research Veterinary Institute, Sofia, Bulgaria*

*^5^ Danish Veterinary and Food Administration, Denmark*

*^6^ University of Copenhagen, Copenhagen, Denmark*

*^7^* *Finnish Institute for Health and Welfare, Finland*

*^8^ Health Protection Surveillance Centre, Dublin, Ireland*

*^9^ Institut Pasteur, Université Paris Cité, Unité des Bactéries pathogènes entériques, Centre National de Référence des Escherichia coli, Shigella et Salmonella, Paris F-75015, France*

*^10^ Santé publique France, France*

*^11^ Robert Koch Institute, Germany*

*^12^ The National Institute of Public Health, Slovenia*

*^13^ National Laboratory of Health, Environment and Food, Slovenia*

*^14^ Public health Agency of Sweden, Sweden*

*^15^ UK Health Security Agency, United Kingdom*

*^16^ Istituto Superiore di Sanità, Italy*

*^17^ Institute of Public Health, Norway*

*^18^ Institute of Hygiene and Medical Microbiology, Medical University Innsbruck, Austria*

*^19^ ECDC fellowship Programme, Public Health Microbiology path (EUPHEM), European Centre for Disease Prevention and Control (ECDC), Stockholm, Sweden*

**Methods**

*Microbiology and whole genome sequencing analysis (Denmark and Austria):*

For genomes sequenced at the Statens Serum Institut in Denmark, genomic DNA was extracted and purified using an automated setup with the MagNa Pure 96 DNA and Viral NA Small Volume kit and according to the DNA Blood SV 2.0 protocol (Roche Diagnostics, Switzerland). Libraries were constructed using the Nextera XT DNA Library Preparation Kit and whole genomes sequencing was performed using 300-cycle kit and the NextSeq 550 platform (Illumina, USA) according to manufacturer protocol. Quality of the sequences were assessed using the in-house pipeline Bifrost (<https://github.com/ssi-dk/bifrost>); Verification of species and contamination check using Bracken v. 1.0.0-1 (<https://github.com/jenniferlu717/Bracken>, <https://doi.org/10.7717/peerj-cs.104>), assembly quality, genome size and adequate sequence depth > 50. Serotypes were determined from genomes using SeqSero v. 1.1.0 (https://doi.org/10.1128/jcm.00323-15, http://denglab.info/SeqSero2) and compared to the predicted serotype from Enterobase (https://enterobase.warwick.ac.uk/) based on multi locus sequence typing (MLST). MLST was determined according to the seven gene MLST scheme for Salmonella enterica (https://doi.org/10.1016/S1567-1348(02)00089-8) using ARIBA v. 1.14.1 for read mapping analysis (https://doi.org/10.1099/mgen.0.000131, https://sanger-pathogens.github.io/ariba/). Core genome MLST (cgMLST) analysis based on the Enterobase S*.* enterica scheme (https://enterobase.warwick.ac.uk/) was used for cluster and outbreak detection. CgMLST analysis and minimum spanning tree were calculated in Bionumerics v. 8.1.1 (bioMérieux, France).

For genomes sequenced at the Danish Veterinary and Food administration, genomic DNA was extracted by Invitrogen Easy-DNA™ gDNA Purification Kit. Libraries were constructed using the Nextera XT DNA Library Preparation Kit and whole genomes sequencing was performed by Miseq system (Ilumina, USA) using 2 x 251 cycle kit Miseq regent kit v3, according to manufacturer protocol.

In Austria, at the Austrian Agency for Health and Food Safety, for extraction of high molecular weight genomic DNA, the isolates were subcultured overnight on modified Lab-Lemco agar (Oxoid) at 37°C followed by DNA purification using HMW MagAttract kit (Qiagen, Hilden, Germany) according to manufacturer’s instructions for Gram negative bacteria.

Whole genome sequencing (WGS) was performed using an Illumina MiSeq platform (Illumina Inc., San Diego, CA, USA). Library preparation was carried out using Nextera XT according to the instructions of the manufacturer (Illumina Inc., San Diego, CA, USA). Raw reads were de novo assembled into draft genomes using SPAdes version 3.15.5 (3). The obtained contigs were filtered for a minimum coverage of 5x and a minimum length of 200 base pairs using SeqSphere+ software v 9.0.3 (4) (Ridom, Münster, Germany). The cgMLST analysis was performed based on the task template of the Ridom SeqSphere+ software v. 9.0.3 (Ridom, Münster, Germany), using 3,002 S. enterica target gene loci. This scheme was used to establish the genotypic relationship between isolates, which was then visualized using a minimum spanning tree (MST). In addition, Sequence Type (ST) and Complex Type (CT) were extracted from WGS sequence data.
