# Supplementary material for: Widespread European outbreaks of Salmonella Enteritidis related to chicken meat from Poland, 2023
Source: Epidemiol Infect. 2026 Sep 24;154:e118. doi: 10.1017/S0950268826102015 (PMC13749103; doi:10.1017/S0950268826102015)
Supplement: Supplementary file 1 [file S0950268826102015sup001.zip › AGES Fragebogen 05 2023 Salmonella Enteritidis ST11 CT13755.docx]

**Fragebogen für Erkrankungen durch *Salmonella* Enteritidis ST11 CT13755**

**05/2023_*Salmonella* Enteritidis_Kärnten**

Datum der Befragung:

| Bei gastrointestinaler Symptomatik | 6 Stunden bis 72 Stunden, längere Inkubationszeiten von bis zu 16 Tagen wurden zwar schon dokumentiert, kommt aber in der Regel selten vor |
| --- | --- |

**Personenbezogene Daten**

| Name | | | EMS ID |
| --- | --- | --- | --- |
| Geburtsdatum | Alter | | Nationalität |
| Wohnadresse: PLZ, Ort, Straße und Haus/Stiege/Tür Nr.: | | | |
| Telefon Nr. | | Geschlecht ☐ Männlich ☐ Weiblich | |
| Beruf/Tätigkeit | | Dienstgeber bzw. Kindergarten/Schule/Klasse | |

Krankheitsverlauf

| Wann hat die Erkrankung begonnen?  *Erkrankungsbeginn ist definiert durch Einsetzen von Durchfall oder Fieber* | | Datum: | | | |
| --- | --- | --- | --- | --- | --- |
| Welche Symptome sind aufgetreten? (Mehrfachangaben möglich)  ☐ Übelkeit ☐ Erbrechen ☐ Bauchschmerzen bzw. Krämpfe ☐ Durchfall  ☐ Fieber über 38°C ☐ Andere Beschwerden:  ☐ Asymptomatisch (keinerlei Krankheitszeichen und daher auch kein Krankheitsbeginn) | | | | | |
| Ende des Durchfalls (Datum) bzw. Ende der Beschwerden: | | | | | |
| War ein Krankenhausaufenthalt erforderlich? | | | ☐ Ja | ☐ Nein |  |
| Name von Krankenhaus | | | | |  |
| Wurden Sie auf eine Intensivstation aufgenommen? | | | ☐ Ja | ☐ Nein |  |
| Aufnahmedatum | Entlassungsdatum | | | |  |

**Fallsuche**

| 1. Hatten Sie in den letzten 3 Tagen vor dem Erkrankungsbeginn Kontakt zu einer Person, die an Durchfall erkrankt ist? | ☐ Ja | ☐ Nein |
| --- | --- | --- |

**Falls ja:** Angaben zu weiteren Personen mit Gastroenteritis-Symptomen die im gleichen Haushalt/Gemeinschaftseinrichtung leben sowie enge Kontaktpersonen im beruflichen oder im privaten (Freizeit) Setting

| Name | Geb.-Datum oder Alter | Erkrankungsbeginn  (Datum und Uhrzeit) | Setting |
| --- | --- | --- | --- |
|  |  |  |  |
|  |  |  |  |
|  |  |  |  |

**Fragen zu Reiseinformationen**

2. Waren Sie in den vergangenen 3 Tagen vor Erkrankungsbeginn auf einer Reise im Ausland (privat oder dienstlich)?

| ☐ Ja | ☐ Nein | Angaben zu Ort und Land: | |
| --- | --- | --- | --- |
| Datum Beginn der Reise: | | |  |
| Datum Ende der Reise: | | |  |

**Fragen zu Veranstaltungen** (Bitte nehmen Sie sich gerne Ihren Kalender oder Handyfotos zur Hilfe)

3. Haben Sie in den vergangenen 3 Tagen vor Erkrankungsbeginn an einer Veranstaltung oder Gruppenreise teilgenommen (zB Feiertag, Geburtstagsfeier, Hochzeit, Betriebsausflug, Catering, …)?

| Veranstaltung | ☐ Ja | ☐ Nein | Ort, Datum: |
| --- | --- | --- | --- |
| Veranstalter | ☐ Ja | ☐ Nein |  |
| Catering | ☐ Ja | ☐ Nein |  |

**Angaben zum Auswärtsessen** (Bitte nehmen Sie sich gerne Ihren Kalender oder Handyfotos zur Hilfe)

4. Haben Sie in den vergangenen 3 Tagen vor Erkrankungsbeginn außer Haus gegessen?

| **Anbieter** | Ja | Nein | **Anbieter** | Ja | Nein |
| --- | --- | --- | --- | --- | --- |
| Restaurant ** Kebab Stand! | ☐ | ☐ | Bäcker / Konditor | ☐ | ☐ |
| Autobahnraststätte | ☐ | ☐ | Familie / Freunde | ☐ | ☐ |
| Lieferservice | ☐ | ☐ | Krankenhaus, Altenheim, Pflegeheim, Betreutes Wohnen | ☐ | ☐ |
| Schule, Kantine | ☐ | ☐ |  |  |  |

**Wenn ja, bitte genauen Datum des Besuchs, Namen des Lokals, Adresse und wenn möglich die verzehrten Speisen bekannt geben:**

| Anbieter | Datum des Besuchs | Lokalname | Adresse | Konsumierte Speisen |
| --- | --- | --- | --- | --- |
| Kebab Stand |  |  |  |  |
| Restaurant |  |  |  |  |
| Autobahnraststätte |  |  |  |  |
| Lieferservice |  |  |  |  |
| Schule, Kantine |  |  |  |  |
| Bäcker / Konditor |  |  |  |  |
| Familie / Freunde |  |  |  |  |
| Krankenhaus, Altenheim, Pflegeheim, Betreutes Wohnen |  |  |  |  |

**Allgemeine Informationen zu Essverhalten**

5. Wer kocht für sie zuhause?

6. Wer geht Lebensmittel einkaufen?

7. Wie viele Personen leben in Ihrem Haushalt? (Anzahl)

**Wie sind Ihre Essgewohnheiten?**

8. Ernähren Sie sich überwiegend oder ausschließlich: **Mehrfach ankreuzen möglich**

| Antwort Möglichkeiten | ERNÄHRUNGSGEWOHNHEITEN |
| --- | --- |
| ☐ Ja ☐ Weiß nicht ☐Nein | Vegan |
| ☐ Ja ☐ Weiß nicht ☐Nein | Vegetarisch |
| ☐ Ja ☐ Weiß nicht ☐Nein | Lactosefrei |
| ☐ Ja ☐ Weiß nicht ☐Nein | Gluten frei |
| ☐ Ja ☐ Weiß nicht ☐Nein | Mit Produkten für Diabetiker |
| ☐ Ja ☐ Weiß nicht ☐Nein | Nuss frei |
| ☐ Ja ☐ Weiß nicht ☐Nein | Kalorienarm/gewichtsreduzierend |
| ☐ Ja ☐ Weiß nicht ☐Nein | Nach einer religiösen Vorschrift (z.B. koscher, Halal) |
| ☐ Ja ☐ Weiß nicht ☐Nein | Kaufen Sie bewusst (nicht notwendigerweise ausschließlich) Bio-Lebensmittel? |
| ☐ Ja ☐ Weiß nicht ☐Nein | Verwenden Sie Nahrungsergänzungsmittel in Pulverform |
| ☐ Ja ☐ Weiß nicht ☐Nein | Verwenden Sie sonstige Nahrungsergänzungsmittel (z.B. Vitamine, Spurenelemente, etc. ) |
| ☐ Ja ☐ Weiß nicht ☐Nein | Haben Sie sich vor Ihrer Erkrankung aus religiösen, gewichtsreduzierenden, medizinischen o.ä. Gründen außergewöhnlich ernährt?  Falls ja, beschreiben Sie diese Ernährungsweise bitte genauer: |

Kaufverhalten

9. Bitte nennen Sie die Quellen, in denen Sie **üblicherweise Lebensmittel einkaufen**

| **Einkaufsmöglichkeit** | **Häufig** | **Selten** | **Nie** | **Adresse der Filiale(n)** |
| --- | --- | --- | --- | --- |
| ADEG | ☐ | ☐ | ☐ |  |
| Billa oder Billa Plus | ☐ | ☐ | ☐ |  |
| Spar, Eurospar, Interspar | ☐ | ☐ | ☐ |  |
| Hofer | ☐ | ☐ | ☐ |  |
| Lidl | ☐ | ☐ | ☐ |  |
| M Preis | ☐ | ☐ | ☐ |  |
| Metro | ☐ | ☐ | ☐ |  |
| Nah&Frisch | ☐ | ☐ | ☐ |  |
| Penny | ☐ | ☐ | ☐ |  |
| Sutterlüty | ☐ | ☐ | ☐ |  |
| Unimarkt | ☐ | ☐ | ☐ |  |
| Andere Supermärkte | ☐ | ☐ | ☐ |  |
| Online Supermarkt **Alfies.at** | ☐ | ☐ | ☐ |  |
| Online Supermarkt **Gurkerl.at** | ☐ | ☐ | ☐ |  |
| Abhofverkauf (Bauernmarkt, Bauernhof, etc.) | ☐ | ☐ | ☐ |  |
| Andere Lebensmittelgeschäfte, Dorfladen, Greißler, etc. | ☐ | ☐ | ☐ |  |
| Anderer Anbieter für Online Lebensmittel Bestellungen mit Lieferung | ☐ | ☐ | ☐ |  |

**Spezifische Lebensmittelanamnese**:

10. Wie häufig in den vergangenen zwei Wochen vor Erkrankungsbeginn haben Sie die folgenden Lebensmittel gegessen?

Bitte wenn möglich auch Angaben zu **Marke/Sorte**, **wo** eingekauft, **wann** eingekauft hinzufügen

| FLEISCH | Öfters | Nie | Ich bin mir nicht sicher | Marke, Einkaufsort |
| --- | --- | --- | --- | --- |
| Rind | ☐ | ☐ | ☐ |  |
| Faschiertes | ☐ | ☐ | ☐ |  |
| Beef Tartar | ☐ | ☐ | ☐ |  |
| Steak od. Filet | ☐ | ☐ | ☐ |  |
| Hamburger | ☐ | ☐ | ☐ |  |
| Anderes Rindprodukt: | ☐ | ☐ | ☐ |  |
| Schwein | ☐ | ☐ | ☐ |  |
| Gemischtes Faschiertes | ☐ | ☐ | ☐ |  |
| Filet, Kotelett od. Medaillons | ☐ | ☐ | ☐ |  |
| Braten | ☐ | ☐ | ☐ |  |
| Schnitzel | ☐ | ☐ | ☐ |  |
| Anderes Schweinefleisch Produkt: | ☐ | ☐ | ☐ |  |
| Lamm | ☐ | ☐ | ☐ |  |
| Huhn | ☐ | ☐ | ☐ |  |
| Grillhuhn, bratfertig | ☐ | ☐ | ☐ |  |
| Brust, Innenfilet | ☐ | ☐ | ☐ |  |
| Oberkeulenfilet | ☐ | ☐ | ☐ |  |
| Keule | ☐ | ☐ | ☐ |  |
| Schnitzel | ☐ | ☐ | ☐ |  |
| Innereien | ☐ | ☐ | ☐ |  |
| Anderes Hühnerfleisch Produkt: | ☐ | ☐ | ☐ |  |
| Pute | ☐ | ☐ | ☐ |  |
| Brust, Innenfilet | ☐ | ☐ | ☐ |  |
| Flügerl, Keule | ☐ | ☐ | ☐ |  |
| Ganz, gebraten | ☐ | ☐ | ☐ |  |
| Anderes Putenfleisch Produkt: | ☐ | ☐ | ☐ |  |
| Ente | ☐ | ☐ | ☐ |  |
| Gans | ☐ | ☐ | ☐ |  |
| Wild | ☐ | ☐ | ☐ |  |
| Anderes Fleisch: | ☐ | ☐ | ☐ |  |

| WURSTWAREN, SCHINKEN & SPECK | Öfters | Nie | Ich bin mir nicht sicher | Marke, Einkaufsort |
| --- | --- | --- | --- | --- |
| Salami vom Schwein, geschnitten | ☐ | ☐ | ☐ |  |
| Milano | ☐ | ☐ | ☐ |  |
| Pfeffer | ☐ | ☐ | ☐ |  |
| Trüffel | ☐ | ☐ | ☐ |  |
| Andere Salami- Sorten vom Schwein: | ☐ | ☐ | ☐ |  |
| Putensalami | ☐ | ☐ | ☐ |  |
| Salami, ganz | ☐ | ☐ | ☐ |  |
| Salami Sticks | ☐ | ☐ | ☐ |  |
| Speck | ☐ | ☐ | ☐ |  |
| Frühstücksspeck | ☐ | ☐ | ☐ |  |
| Bauchspeck | ☐ | ☐ | ☐ |  |
| Speckwürfel | ☐ | ☐ | ☐ |  |
| Schinkenspeck | ☐ | ☐ | ☐ |  |
| Karreespeck | ☐ | ☐ | ☐ |  |
| Andere Speck-Sorten: | ☐ | ☐ | ☐ |  |
| Grammelschmalz | ☐ | ☐ | ☐ |  |
| Andere Schmalz-Sorten | ☐ | ☐ | ☐ |  |
| Grammeln | ☐ | ☐ | ☐ |  |
| Wurstaufschnitt | ☐ | ☐ | ☐ |  |
| Schinken | ☐ | ☐ | ☐ |  |
| Schwarzwälder- | ☐ | ☐ | ☐ |  |
| Toast- | ☐ | ☐ | ☐ |  |
| Farmer- | ☐ | ☐ | ☐ |  |
| Bein- | ☐ | ☐ | ☐ |  |
| Andere Schinken-Sorten: | ☐ | ☐ | ☐ |  |
| Wurstspezialitäten | ☐ | ☐ | ☐ |  |
| Prosciutto Crudo/Cotto | ☐ | ☐ | ☐ |  |
| Ibéricoschinken | ☐ | ☐ | ☐ |  |
| Jamon Serrano / Schinken | ☐ | ☐ | ☐ |  |
| Rinderschinken (Bresaola) | ☐ | ☐ | ☐ |  |
| Mortadella | ☐ | ☐ | ☐ |  |
| Chorizo | ☐ | ☐ | ☐ |  |
| (Pariser)Kranzl | ☐ | ☐ | ☐ |  |
| Extrawurst | ☐ | ☐ | ☐ |  |
| Kalbspariser | ☐ | ☐ | ☐ |  |
| Käsewurst | ☐ | ☐ | ☐ |  |
| Leberstreichwurst | ☐ | ☐ | ☐ |  |
| Bauernpresswurst, Sülze/Aspik | ☐ | ☐ | ☐ |  |
| Andere Wurst-Sorten: | ☐ | ☐ | ☐ |  |
| Brühwurst | ☐ | ☐ | ☐ |  |
| Frankfurter | ☐ | ☐ | ☐ |  |
| Debreziner | ☐ | ☐ | ☐ |  |
| Weißwurst | ☐ | ☐ | ☐ |  |
| Andere Brühwurst-Sorten: | ☐ | ☐ | ☐ |  |
| Grillwürstel | ☐ | ☐ | ☐ |  |
| Rostbratwürstel | ☐ | ☐ | ☐ |  |
| Berner | ☐ | ☐ | ☐ |  |
| Käsekrainer | ☐ | ☐ | ☐ |  |
| Andere Grillwürstel-Sorten: | ☐ | ☐ | ☐ |  |
| Dauerwurst | ☐ | ☐ | ☐ |  |
| Kantwurst | ☐ | ☐ | ☐ |  |
| Wiesbauer | ☐ | ☐ | ☐ |  |
| Andere Dauerwurst Sorten: | ☐ | ☐ | ☐ |  |
| Knabberwurst |  |  |  |  |
| Landjäger, Hauswürstel | ☐ | ☐ | ☐ |  |
| Kaminwurzel |  |  |  |  |
| Cabanossi, Salanettis | ☐ | ☐ | ☐ |  |
| Andere Knabberwurst-Sorten: | ☐ | ☐ | ☐ |  |
| Sonstiges aus der Feinkost-Theke: | ☐ | ☐ | ☐ |  |

| MILCH & MILCHPRODUKTE | Öfters | Nie | Ich bin mir nicht sicher | Marke, Einkaufsort |
| --- | --- | --- | --- | --- |
| Milch | ☐ | ☐ | ☐ |  |
| Frischmilch Vollmilch (pasteurisiert) | ☐ | ☐ | ☐ |  |
| Frischmilch Halbfettmilch / Leicht (pasteurisiert) | ☐ | ☐ | ☐ |  |
| Frischmilch Vollmilch länger frisch (pasteurisiert) | ☐ | ☐ | ☐ |  |
| Laktosefreie Vollmilch | ☐ | ☐ | ☐ |  |
| Laktosefreie Halbfettmilch | ☐ | ☐ | ☐ |  |
| H-Milch Vollmilch | ☐ | ☐ | ☐ |  |
| H-Milch Leicht/Fettarm | ☐ | ☐ | ☐ |  |
| Milchpulver | ☐ | ☐ | ☐ |  |
| Buttermilch | ☐ | ☐ | ☐ |  |
| Andere Milch-Sorten: | ☐ | ☐ | ☐ |  |
| Rohmilch | ☐ | ☐ | ☐ |  |
| Sojadrink | ☐ | ☐ | ☐ |  |
| Haferdrink | ☐ | ☐ | ☐ |  |
| Reisdrink | ☐ | ☐ | ☐ |  |
| Andere Milch-Ersatz-Drink Sorten: | ☐ | ☐ | ☐ |  |
| Butter | ☐ | ☐ | ☐ |  |
| Topfen | ☐ | ☐ | ☐ |  |
| Jogurt | ☐ | ☐ | ☐ |  |
| Schlagobers | ☐ | ☐ | ☐ |  |
| Sauerrahm/Crème fraiche | ☐ | ☐ | ☐ |  |
| Kakao | ☐ | ☐ | ☐ |  |
| Eiskrem | ☐ | ☐ | ☐ |  |
| Sonstige Milchprodukte: | ☐ | ☐ | ☐ |  |

| EIER ODER EI-ENTHALTENDE LEBENSMITTEL | Öfters | Nie | Ich bin mir nicht sicher | Marke, Einkaufsort |
| --- | --- | --- | --- | --- |
| Eier | ☐ | ☐ | ☐ |  |
| Bodenhaltung | ☐ | ☐ | ☐ |  |
| Freilandhaltung | ☐ | ☐ | ☐ |  |
| Bio-Eier | ☐ | ☐ | ☐ |  |
| Weichgekochte Eier | ☐ | ☐ | ☐ |  |
| Spiegeleier/Omelette | ☐ | ☐ | ☐ |  |
| Ei-Ersatz | ☐ | ☐ | ☐ |  |
| Ei Aufstrich | ☐ | ☐ | ☐ |  |
| Ei Salat | ☐ | ☐ | ☐ |  |
| Eier-Likör | ☐ | ☐ | ☐ |  |
| Mayonnaise gekauft, selbstgemacht | ☐ | ☐ | ☐ |  |
| Tiramisu | ☐ | ☐ | ☐ |  |
| Sonstige Ei-enthaltenden Speisen: | ☐ | ☐ | ☐ |  |

| KÄSE | Öfters | Nie | Ich bin mir nicht sicher | Marke, Einkaufsort |
| --- | --- | --- | --- | --- |
| Weißschmimmelkäse | ☐ | ☐ | ☐ |  |
| Camembert | ☐ | ☐ | ☐ |  |
| Brie | ☐ | ☐ | ☐ |  |
| Blauschimmelkäse | ☐ | ☐ | ☐ |  |
| Gorgonzola | ☐ | ☐ | ☐ |  |
| Roquefort | ☐ | ☐ | ☐ |  |
| Frischkäse | ☐ | ☐ | ☐ |  |
| Hüttenkäse | ☐ | ☐ | ☐ |  |
| Ricotta | ☐ | ☐ | ☐ |  |
| Mascarpone | ☐ | ☐ | ☐ |  |
| Streich- oder Schmelzkäse | ☐ | ☐ | ☐ |  |
| Ziegenkäse | ☐ | ☐ | ☐ |  |
| Schafskäse/ Feta | ☐ | ☐ | ☐ |  |
| Mozzarella, gerieben oder ganz | ☐ | ☐ | ☐ |  |
| Schnittkäse | ☐ | ☐ | ☐ |  |
| Gouda | ☐ | ☐ | ☐ |  |
| Edamer | ☐ | ☐ | ☐ |  |
| Butterkäse | ☐ | ☐ | ☐ |  |
| Tilsiter | ☐ | ☐ | ☐ |  |
| Andere Schnittkäse Sorten: | ☐ | ☐ | ☐ |  |
| Raclette | ☐ | ☐ | ☐ |  |
| Haloumi | ☐ | ☐ | ☐ |  |
| Quargel | ☐ | ☐ | ☐ |  |
| Cheddar | ☐ | ☐ | ☐ |  |
| Hartkäse | ☐ | ☐ | ☐ |  |
| Appenzeller | ☐ | ☐ | ☐ |  |
| Grüyer | ☐ | ☐ | ☐ |  |
| Emmentaler | ☐ | ☐ | ☐ |  |
| Parmesan | ☐ | ☐ | ☐ |  |
| Andere Hartkäse Sorten: | ☐ | ☐ | ☐ |  |
| Käse, gekauft direkt bei einer Käserei/Hof | ☐ | ☐ | ☐ |  |
| Käse, gekauft direkt bei Marktstand oder Spezialitätengeschäft | ☐ | ☐ | ☐ |  |
| Käse, vom Ausland mitgebracht: | ☐ | ☐ | ☐ |  |
| Andere Käse Sorten: | ☐ | ☐ | ☐ |  |

| FERTIGSALATE, AUCH ALS BROTAUFSTRICH | Öfters | Nie | Ich bin mir nicht sicher | Marke, Einkaufsort |
| --- | --- | --- | --- | --- |
| Couscous-Salat | ☐ | ☐ | ☐ |  |
| Fleischsalat (Wurstsalat) | ☐ | ☐ | ☐ |  |
| Geflügelsalat | ☐ | ☐ | ☐ |  |
| Heringssalat | ☐ | ☐ | ☐ |  |
| Kartoffelsalat | ☐ | ☐ | ☐ |  |
| Krabbensalat | ☐ | ☐ | ☐ |  |
| Krautsalat | ☐ | ☐ | ☐ |  |
| Meeresfrüchtesalat (z.B. Shrimpscocktail) | ☐ | ☐ | ☐ |  |
| Nudelsalat | ☐ | ☐ | ☐ |  |
| Spargelsalat | ☐ | ☐ | ☐ |  |
| Sonstige Salat-Sorten: | ☐ | ☐ | ☐ |  |

| FRISCHER FISCH & MEERESFRÜCHTE | Öfters | Nie | Ich bin mir nicht sicher | Marke, Einkaufsort |
| --- | --- | --- | --- | --- |
| Matjesfilet, Hering eingelegt | ☐ | ☐ | ☐ |  |
| Lachs | ☐ | ☐ | ☐ |  |
| Räucherlachs, in Scheiben | ☐ | ☐ | ☐ |  |
| Anderer frischer gekaufter Fisch (ganz, Filet etc.): | ☐ | ☐ | ☐ |  |
| Sushi | ☐ | ☐ | ☐ |  |
| Austern | ☐ | ☐ | ☐ |  |
| Muscheln | ☐ | ☐ | ☐ |  |
| Andere Meeresfrüchte (Hummer, Garnelen, Shrimps, Krabben, Tintenfisch etc.): | ☐ | ☐ | ☐ |  |
| Geräucherter oder getrockneter Fisch (Steckerlfisch, Makrele, etc.) | ☐ | ☐ | ☐ |  |
| Andere Fisch & Meeresfrüchte-Sorten: | ☐ | ☐ | ☐ |  |

| TIEFKÜHLKOST | Öfters | Nie | Ich bin mir nicht sicher | Marke, Einkaufsort |
| --- | --- | --- | --- | --- |
| TK Gemüse | ☐ | ☐ | ☐ |  |
| Spinat | ☐ | ☐ | ☐ |  |
| Fisolen | ☐ | ☐ | ☐ |  |
| Rotkraut | ☐ | ☐ | ☐ |  |
| Kohlsprossen | ☐ | ☐ | ☐ |  |
| Andere TK Gemüse-Sorten: | ☐ | ☐ | ☐ |  |
| TK Beeren (Erdbeeren, Himbeeren, Beerenmischung etc.) | ☐ | ☐ | ☐ |  |
| Erdbeeren | ☐ | ☐ | ☐ |  |
| Himbeeren | ☐ | ☐ | ☐ |  |
| Heidelbeeren | ☐ | ☐ | ☐ |  |
| Beerenmischung | ☐ | ☐ | ☐ |  |
| Andere TK Beeren-Sorten: | ☐ | ☐ | ☐ |  |
| TK Kräuter | ☐ | ☐ | ☐ |  |
| Petersilie | ☐ | ☐ | ☐ |  |
| Schnittlauch | ☐ | ☐ | ☐ |  |
| Andere TK Kräuter Sorten: | ☐ | ☐ | ☐ |  |
| TK Fertiggerichte | ☐ | ☐ | ☐ |  |
| Pizza | ☐ | ☐ | ☐ |  |
| Hühner Nuggets | ☐ | ☐ | ☐ |  |
| Andere TK-Fertiggerichte Sorten: | ☐ | ☐ | ☐ |  |
| TK Fisch | ☐ | ☐ | ☐ |  |
| Fischstäbchen | ☐ | ☐ | ☐ |  |
| Andere TK Fisch-Sorten: | ☐ | ☐ | ☐ |  |
| TK Garnelen | ☐ | ☐ | ☐ |  |
| Ofengarnelen | ☐ | ☐ | ☐ |  |
| Andere TK Garnelen Sorten: | ☐ | ☐ | ☐ |  |
| Andere TK Produkte: | ☐ | ☐ | ☐ |  |

| GEMÜSE | Öfters | Nie | Ich bin mir nicht sicher | Marke, Einkaufsort |
| --- | --- | --- | --- | --- |
| Sprossen und Keimlinge | ☐ | ☐ | ☐ |  |
| Sojabohnensprossen | ☐ | ☐ | ☐ |  |
| Mungo-Bohnenkeimlinge | ☐ | ☐ | ☐ |  |
| Kresse | ☐ | ☐ | ☐ |  |
| Selbstgezogene Sprossen, Keimlinge, Gräser etc. | ☐ | ☐ | ☐ |  |
| Andere Sprossen Sorten: | ☐ | ☐ | ☐ |  |
| Kleine Tomaten | ☐ | ☐ | ☐ |  |
| Cherrytomaten, mit Rispe | ☐ | ☐ | ☐ |  |
| Cherrytomaten, ohne Rispe | ☐ | ☐ | ☐ |  |
| Große Tomaten | ☐ | ☐ | ☐ |  |
| Rispentomaten | ☐ | ☐ | ☐ |  |
| Fleischtomaten, ohne Rispe | ☐ | ☐ | ☐ |  |
| Getrocknete Tomaten | ☐ | ☐ | ☐ |  |
| Wurzelgemüse | ☐ | ☐ | ☐ |  |
| Karotten | ☐ | ☐ | ☐ |  |
| Radieschen | ☐ | ☐ | ☐ |  |
| Kartoffeln | ☐ | ☐ | ☐ |  |
| Pastinake | ☐ | ☐ | ☐ |  |
| Knollensellerie | ☐ | ☐ | ☐ |  |
| Andere Würzelgemüse-Sorten: | ☐ | ☐ | ☐ |  |
| Blattgemüse | ☐ | ☐ | ☐ |  |
| Spinat | ☐ | ☐ | ☐ |  |
| Kopfsalat | ☐ | ☐ | ☐ |  |
| Eisbergsalat | ☐ | ☐ | ☐ |  |
| Endivien |  |  |  |  |
| Rucola | ☐ | ☐ | ☐ |  |
| Andere Blattgemüse-Sorten: | ☐ | ☐ | ☐ |  |
| Avocado | ☐ | ☐ | ☐ |  |
| Salatgurke | ☐ | ☐ | ☐ |  |
| Paprika | ☐ | ☐ | ☐ |  |
| Frühlingszwiebel | ☐ | ☐ | ☐ |  |
| Porree/Lauch | ☐ | ☐ | ☐ |  |
| Fenchel | ☐ | ☐ | ☐ |  |
| Andere Gemüse-Sorten: | ☐ | ☐ | ☐ |  |

| FRISCHES OBST | Öfters | Nie | Ich bin mir nicht sicher | Marke, Einkaufsort |
| --- | --- | --- | --- | --- |
| Äpfel | ☐ | ☐ | ☐ |  |
| Elstar | ☐ | ☐ | ☐ |  |
| Braeburn | ☐ | ☐ | ☐ |  |
| Gala | ☐ | ☐ | ☐ |  |
| Andere Apfel-Sorte: | ☐ | ☐ | ☐ |  |
| Birnen | ☐ | ☐ | ☐ |  |
| Xenia | ☐ | ☐ | ☐ |  |
| Abate Fetel | ☐ | ☐ | ☐ |  |
| Kaiser Alexander | ☐ | ☐ | ☐ |  |
| Andere Birnen Sorte: | ☐ | ☐ | ☐ |  |
| Melone | ☐ | ☐ | ☐ |  |
| Wassermelone | ☐ | ☐ | ☐ |  |
| Zuckermelone | ☐ | ☐ | ☐ |  |
| Andere Melonen Sorte: | ☐ | ☐ | ☐ |  |
| Bananen | ☐ | ☐ | ☐ |  |
| Beeren | ☐ | ☐ | ☐ |  |
| Erdbeeren | ☐ | ☐ | ☐ |  |
| Himbeeren | ☐ | ☐ | ☐ |  |
| Blaubeeren | ☐ | ☐ | ☐ |  |
| Andere Beeren Sorte: | ☐ | ☐ | ☐ |  |
| Ananas | ☐ | ☐ | ☐ |  |
| Mango | ☐ | ☐ | ☐ |  |
| Kiwi | ☐ | ☐ | ☐ |  |
| Orangen | ☐ | ☐ | ☐ |  |
| Mandarinen | ☐ | ☐ | ☐ |  |
| Trauben (grün/rot) | ☐ | ☐ | ☐ |  |
| Marille | ☐ | ☐ | ☐ |  |
| Nektarine/Pfirsich | ☐ | ☐ | ☐ |  |
| Pflaume, Zwetschge | ☐ | ☐ | ☐ |  |
| Feigen | ☐ | ☐ | ☐ |  |
| Andere Obst Sorten: | ☐ | ☐ | ☐ |  |

| FRUCHTSÄFTE, SMOOTHIES & MILCHSHAKES | Öfters | Nie | Ich bin mir nicht sicher | Marke, Einkaufsort |
| --- | --- | --- | --- | --- |
| Fruchtshakes, Smoothies, Milchshakes | ☐ | ☐ | ☐ |  |
| Frisch gepresster Apfel-/Orangensaft | ☐ | ☐ | ☐ |  |
| Anderer frisch gepresster Frucht-/Gemüsesaft (nicht erhitzt) | ☐ | ☐ | ☐ |  |
| Orangensaft; als Direktsaft oder aus Konzentrat hergestellt | ☐ | ☐ | ☐ |  |
| Apfelsaft; als Direktsaft oder aus Konzentrat hergestellt | ☐ | ☐ | ☐ |  |
| Anderer Obstsaft; als Direktsaft oder aus Konzentrat hergestellt | ☐ | ☐ | ☐ |  |
| Gemüsesaft, als Direktsaft oder aus Konzentrat hergestellt | ☐ | ☐ | ☐ |  |
| Fruchtnektar | ☐ | ☐ | ☐ |  |
| Verdünn-Saft | ☐ | ☐ | ☐ |  |
| Andere Saft Sorten: | ☐ | ☐ | ☐ |  |
