# Supplementary material for: Widespread European outbreaks of Salmonella Enteritidis related to chicken meat from Poland, 2023
Source: Epidemiol Infect. 2026 Sep 24;154:e118. doi: 10.1017/S0950268826102015 (PMC13749103; doi:10.1017/S0950268826102015)
Supplement: Supplementary file 1 [file S0950268826102015sup001.zip › S. enteritidis_questionnaire_DK.docx]

**Salmonella Enteritidis ST11	FUD2239**

**Hypotesegenererende interviews**

Interview foretaget af _________________________ Dato for interview____/____/____

Bemærkninger:

Hvem interviewes? Patienten selv ☐ En anden ☐ hvem________________

**1. PATIENT INFORMATION**

1.1 Fornavn: ______________________________

1.2 Efternavn: ______________________________________

1.3 Telefonnummer: _________________________

1.4 Køn: Kvinde ☐ Mand ☐

1.5 Alder: _____ år	1.6 Prøvedato: ____ /____ /________

_________________________________________________________________________________________________________________

**Goddag mit navn er XX, taler jeg med XX? Jeg ringer fra Statens Serum Institut. Jeg ringer fordi du/dit barn har haft en infektion med Salmonella. Det viser sig, at der er flere, der har været syge med den samme type af salmonella og vi mistænker, at I er blevet smittet af den samme fødevare. Jeg vil derfor høre dig, om jeg må stille nogle spørgsmål angående din/(dit barns) sygdom og hvad du/(han/hun) spiser?**

*(Hvis spørgeskemaet gælder et barn spørges ”dit barn” eller barnets navn i stedet for du/dig under hele interviewet)*

**2. SYGDOMSHISTORIE**

2.1 Hvilken dato fik du de første symptomer på sygdom? ____/____/____ dd/mm/åå

2.2 Er du stadig syg med salmonella?	Ja ☐	Nej ☐	Usikker ☐

2.2.1 Hvis nej, hvor mange dage var du syg i alt? _________ dage

2.3 Har du haft nogle af følgende symptomer?

| **Symptom** | **Ja** | **Nej** | **Usikker** | **Varighed** |
| --- | --- | --- | --- | --- |
| Diarré |  |  |  |  |
| Blodig diarré |  |  |  |  |
| Mavesmerter |  |  |  |  |
| Kvalme |  |  |  |  |
| Opkast |  |  |  |  |
| Feber |  |  |  |  |
| Ledsmerter |  |  |  |  |
| Hovedpine |  |  |  |  |
| Træthed |  |  |  |  |
| Andet |  |  |  |  |

2.3.1 Andet symptom, beskriv _______________________________________________________________

2.4 Var du indlagt pga din sygdom?	Ja ☐	Nej ☐	Hospital___________________________

2.4.1 Dato for indlæggelse ____/____/____	2.4.2 Dato for udskrivelse ____/____/____	*eller* fortsat indlagt ☐

2.5 Lider du af nogen andre sygdomme?	Ja ☐	Nej ☐	Ukendt ☐

2.5.1 Hvis ja, hvad _____________________________________________________________

2.6 Har du selv nogen idé om, hvad du er blevet syg af?	Ja ☐	Nej ☐

2.6.1. Hvis ja, beskriv______________________________________________________________________________________

2.7 Kender du andre, der har været syge med samme symptomer?	Ja ☐	Nej ☐

2.7.1 Hvis ja, beskriv:

Hvem? ________________________________________________________________________________________

Hvornår syg? ___________________________________________________________________________________

Fælles måltider eller begivenheder? _________________________________________________________________

______________________________________________________________________________________________

2.8 Hvor mange og hvem bor i din hustand?

________ personer i hustand inkl patienten

Beskriv: ________________________________________________________________________________________

2.9 Hvad arbejder du med? (Går barnet i institution/skole?)

________________________________________________________________________________________

3.0 Hvor ligger din arbejdsplads? (hvis barn navn og adresse på institution/skole)

________________________________________________________________________________________

**4. REJSEHISTORIE**

4.1 Har du rejst udenlands med overnatning i de **14 DAGE** inden du begyndte at blive syg?

Ja ☐	Nej ☐

4.1.1 Hvis ja, hvor (lande) ________________________________________________________________

4.1.2 Hvilke byer _________________________________________________________________________

4.2.3 Navn på hotel _______________________________________________________________________

4.1.4 Dato for rejse:	Afrejse	____/____/____	Hjemkomst	____/____/____

4.1.5 Var rejsen ”all inclusive”? Ja ☐	Nej ☐

4.1.6. Rejste du med rejseselskab? Ja ☐	Nej ☐

Hvis ja, hvilket rejseselskab? ­­­­­­­­­­­­­­­­­­­­_____________________________________________

**HVIS PATIENTEN HAR VÆRET UDE AT REJSE AFSLUTTES INTERVIEWET HER! Spring da til allersidste spørgsmål om kontakt.**

4.2 Har du rejst indenfor Danmark med overnatning i de **7 DAGE** før du blev syg (fx besøg hos familie eller forretningsrejse)?

Ja ☐	Nej ☐

4.2.1 Hvis ja, hvilket land(e) ________________________________________________________________

4.2.2 Dato for rejse:	Afrejse	____/____/____	Hjemkomst	____/____/____

4.3 Har du deltaget i nogen arrangementer eller fester i de **7 dage** inden du blev syg (fx sportsstævne, en konference eller en familiefest)

Ja ☐	Nej ☐

4.3.1 Hvis ja, beskriv________________________________________________________________

**5. INDKØBSSTEDER & MADVANER**

De næste spørgsmål handler om indkøbssteder og madvaner.

5.1 I hvilke af følgende butikker har du (eller andre i din hustand) købt madvarer i de **14 dage** inden sygdommen begyndte? Har I handlet i…

| **Supermarked/butik** | **Ja** | **Nej** | **Måske** | **Hvor ligger butikken?**  (Adresse eller beliggenhed) |
| --- | --- | --- | --- | --- |
| Superbrugsen |  |  |  |  |
| Daglibrugsen |  |  |  |  |
| Kvickly |  |  |  |  |
| Fakta |  |  |  |  |
| Irma |  |  |  |  |
| Netto |  |  |  |  |
| Føtex |  |  |  |  |
| Bilka |  |  |  |  |
| Meny |  |  |  |  |
| Rema1000 |  |  |  |  |
| Aldi |  |  |  |  |
| Spar |  |  |  |  |
| LIDL |  |  |  |  |
| Lokal grønthandler |  |  |  |  |
| Lokal slagter (eller slagter I supermarked? |  |  |  |  |
| Lokal købmand |  |  |  |  |
| Lokal ostehandler |  |  |  |  |
| Gårdbutik eller lign |  |  |  |  |
| Andet f.eks. marked |  |  |  |  |

5.1.1 Hvis anden butik, hvor__________________________________________________________

5.1.2 Hvis du skulle prioritere, de butikker du lige har nævnt, at du handler i, hvor handler du så mest?

Butik 1: ___________________________________________________

Butik 2: ___________________________________________________

Butik 3: ___________________________________________________

5.2 Får du leveret mad hjem fx gennem en madordning eller et firma som fx nemlig.com eller Årstiderne?	Ja ☐	Nej ☐

5.2.1 Hvis ja, hvorfra_______________________________________________ 5.2.2 Hvor ofte? ____________________________

5.3 Er du vegetar?	Ja ☐	Nej ☐

5.3.1 Hvis ja, hvordan definerer du det (spiser du aldrig kød? hvad med fjerkræ, æg eller fisk?)

_________________________________________________________________________________

5.4 Er der nogle fødevaregrupper, du prøver helt at undgå, fx. på grund af allergi eller af kulturelle årsager?

Ja ☐	Nej ☐

5.4.1 Hvis ja - beskriv

___________________________________________________________________________________________

5.5 Spiser du nogle typer af kosttilskud fx vitaminpiller

Ja ☐	Nej ☐

5.5.1 Hvis ja - beskriv

___________________________________________________________________________________________

5.6 Har du spist eller drukket nogen former for proteindrik eller proteinpulver i de **7 dage** før du blev syg fx i forbindelse med træning eller genoptræning?

Ja ☐	Nej ☐	(gå til spm 7)

5.6.1 Hvis ja – beskriv ______________________________________________________________________________

5.7 Nu vil jeg spørge ind til, hvad du kan huske, du har spist (både til morgenmad, frokost, mellemmåltid og aftensmad) i de **3 dage** inden du blev syg? Her kan det godt være en fordel, hvis du har en kalender ved hånden, så du bedre kan huske de specifikke dage. Så hvis vi starter med det første du spiser om morgenen….

**Dagen før symtomdebut. Dato	______dag	den** ___/___/___	*(skriv evt. videre på bagsiden)*

**Morgenmad**	spist: hjemme ☐	ude ☐ hvor______________________________

_______________________________________________________________________________________________________________

_______________________________________________________________________________________________________________

_______________________________________________________________________________________________________________

**Frokost**	spist: hjemme ☐	ude ☐ hvor______________________________

_______________________________________________________________________________________________________________

_______________________________________________________________________________________________________________

_______________________________________________________________________________________________________________

**Aftensmad**	spist: hjemme ☐	ude ☐ hvor______________________________

_______________________________________________________________________________________________________________

_______________________________________________________________________________________________________________

_______________________________________________________________________________________________________________

**Andet (mellemmåltid, frugt, snacks)**

_______________________________________________________________________________________________________________

_______________________________________________________________________________________________________________

*(skriv evt. videre på bagsiden)*

**2 dage før symtomdebut. Dato	______dag	den** ___/___/___

**Morgenmad**	spist: hjemme ☐	ude ☐ hvor______________________________

_______________________________________________________________________________________________________________

_______________________________________________________________________________________________________________

_______________________________________________________________________________________________________________

**Frokost**	spist: hjemme ☐	ude ☐ hvor______________________________

_______________________________________________________________________________________________________________

_______________________________________________________________________________________________________________

_______________________________________________________________________________________________________________

**Aftensmad**	spist: hjemme ☐	ude ☐ hvor______________________________

_______________________________________________________________________________________________________________

_______________________________________________________________________________________________________________

_______________________________________________________________________________________________________________

**Andet (mellemmåltid, frugt, snacks)**

_______________________________________________________________________________________________________________

_______________________________________________________________________________________________________________

*(skriv evt. videre på bagsiden)*

**3 dage før symtomdebut. Dato	______dag	den** ___/___/___

**Morgenmad**	spist: hjemme ☐	ude ☐ hvor______________________________

_______________________________________________________________________________________________________________

_______________________________________________________________________________________________________________

_______________________________________________________________________________________________________________

**Frokost**	spist: hjemme ☐	ude ☐ hvor______________________________

_______________________________________________________________________________________________________________

_______________________________________________________________________________________________________________

_______________________________________________________________________________________________________________

**Aftensmad**	spist: hjemme ☐	ude ☐ hvor______________________________

_______________________________________________________________________________________________________________

_______________________________________________________________________________________________________________

_______________________________________________________________________________________________________________

**Andet (mellemmåltid, frugt, snacks)**

_______________________________________________________________________________________________________________

_______________________________________________________________________________________________________________

*(skriv evt. videre på bagsiden)*

**6. SPISE UDE**

6.1 Har du spist nogle af følgende steder udenfor hjemmet i de **7 dage** før du blev syg?

| **Spisested** | **Ja** | **Nej** | **Måske** | **Hvis ja, hvornår?** | **Hvad hedder og hvor ligger stedet?** (navn/adresse/beliggenhed) | **Hvad spiste du?** |
| --- | --- | --- | --- | --- | --- | --- |
| Restaurant |  |  |  | ____/____/____ |  |  |
| Café |  |  |  | ____/____/____ |  |  |
| Kantine |  |  |  | ____/____/____ |  |  |
| Skole |  |  |  | ____/____/____ |  |  |
| Daginstitution |  |  |  | ____/____/____ |  |  |
| Plejehjem |  |  |  | ____/____/____ |  |  |
| Hospital |  |  |  | ____/____/____ |  |  |
| I forbindelse med sport fx klub, sportshal el. lign |  |  |  | ____/____/____ |  |  |
| I forbindelse med transport i fx fly, tog, færge |  |  |  | ____/____/____ |  |  |
| Pølsevogn |  |  |  | ____/____/____ |  |  |
| Andet |  |  |  | ____/____/____ |  |  |

6.1.1 Hvis andet spise-ude sted, hvor__________________________________________________________

6.2 Har du spist noget take-away mad –altså færdiglavet mad man tager med hjem fra nogle af følgende steder i de **7 dage** før du blev syg?

| **Take-away sted** | **Ja** | **Nej** | **Usikker** | **Hvis ja, hvornår?** | **Hvad hedder og hvor ligger stedet?** (navn/adresse/beliggenhed) | **Hvad spiste du?** |
| --- | --- | --- | --- | --- | --- | --- |
| Pizzaria |  |  |  | ____/____/____ |  |  |
| Grill-bar |  |  |  | ____/____/____ |  |  |
| Burgerbar fx McDonalds el Burgerking |  |  |  | ____/____/____ |  |  |
| Kebab/shawarma-bar |  |  |  | ____/____/____ |  |  |
| Sandwichbar |  |  |  | ____/____/____ |  |  |
| Delikatesseafd fra fx supermarked |  |  |  | ____/____/____ |  |  |
| Færdigret fra fx køle/frost afd i supermarked, (fx lasagne el pizza) |  |  |  | ____/____/____ |  |  |
| Kiosk |  |  |  | ____/____/____ |  |  |
| Tankstation |  |  |  | ____/____/____ |  |  |
| Andet |  |  |  | ____/____/____ |  |  |

6.2.1 Hvis andet take-away sted, hvor__________________________________________________________

**7. FØDEVARER – KØD OG KØDPÅLÆG**

De næste spørgsmål handler om, hvilke fødevarer du har spist i de 7 dage op til du begyndte at blive syg. Jeg ved godt, at det kan være meget svært at huske, så jeg har fire svarkategorier, der kan hjælpe dig: *Helt sikkert ja, Sandsynligvis ja, Sandsynligvis nej og Helt sikkert nej*. De første spørgsmål handler om forskellige typer kød og fjerkræ.

7.1 Har du i de **7 dage**, inden du blev syg, spist oksekød? Har du spist…

|  | **Ja** | **Sand-synlig-vis ja** | **Sandsynligvis nej** | **Nej** | **Hvis ja eller sandsynligvis ja: Hvilket produkt var det?**  (mærke, dansk, økologisk, frossent?) | **Hvor var det købt?** |
| --- | --- | --- | --- | --- | --- | --- |
| Hakket oksekød |  |  |  |  |  |  |
| Oksebøffer |  |  |  |  |  |  |
| Oksesteg |  |  |  |  |  |  |
| Oksetern eller stykker |  |  |  |  |  |  |
| Kebab |  |  |  |  |  |  |
| Andet oksekød |  |  |  |  |  |  |

7.1.1. Hvis andet oksekød, hvad_______________________________________________________________________________

7.2 Har du spist nogle af følgende typer fjerkræ i de **7 dage** før du blev syg? Har du spist…

|  | **Ja** | **Sand-synlig-vis ja** | **Sandsynligvis nej** | **Nej** | **Hvis ja eller sandsynligvis ja: Hvilket produkt var det?**  (mærke, dansk, økologisk, frossent?) | **Hvor var det købt?** |
| --- | --- | --- | --- | --- | --- | --- |
| Hel kylling |  |  |  |  |  |  |
| Kylling-filetter/bryst |  |  |  |  |  |  |
| Kyllingelår |  |  |  |  |  |  |
| Kalkun |  |  |  |  |  |  |
| Gås |  |  |  |  |  |  |
| And |  |  |  |  |  |  |

7.3 Har du spist nogle af følgende typer svinekød i de **7 dage** før du blev syg? Har du spist…

|  | **Ja** | **Sand-synlig-vis ja** | **Sandsynligvis nej** | **Nej** | **Hvis ja eller sandsynligvis ja: Hvilket produkt var det?**  (mærke, dansk, økologisk) | **Hvor var det købt?** |
| --- | --- | --- | --- | --- | --- | --- |
| Svinesteg, fx skinke, hamburgerryg |  |  |  |  |  |  |
| Flæskesteg eller ribbensteg |  |  |  |  |  |  |
| Svinekotelletter |  |  |  |  |  |  |
| Svinemørbrad |  |  |  |  |  |  |
| Revelsben el spareribs |  |  |  |  |  |  |
| Hakket kalv og flæsk |  |  |  |  |  |  |
| Hakket svinekød |  |  |  |  |  |  |
| Medisterpølse |  |  |  |  |  |  |
| Pølser (fx røde eller wiener) |  |  |  |  |  |  |
| Bacon |  |  |  |  |  |  |
| Skinketern/  -strimler |  |  |  |  |  |  |
| Pulled pork |  |  |  |  |  |  |
| Andet svinekød |  |  |  |  |  |  |

7.3.1 Hvis andet svinekød, hvad_______________________________________________________________________________

7.4 Har du i de **7 dage** før du blev syg spist noget svinekød, der var råt eller utilstrækkelig gennemstegt fx smagt på fars til frikadeller eller spist en lyserød svinekotelet?

Ja ☐	Nej ☐

7.4.1 Hvis ja, beskriv _________________________________________________________________________________

7.5 Har du spist nogle af følgende typer pålæg i de **7 dage** før du blev syg? Har du spist…

| **Pålæg** | **Ja** | **Sand-synlig-vis ja** | **Sandsynligvis nej** | **Nej** | **Hvis ja eller sandsynligt: Hvilket produkt var det?** (mærke, dansk, skiveskåret) | **Hvor var det købt?** |
| --- | --- | --- | --- | --- | --- | --- |
| Skinke i skiver (kogt) |  |  |  |  |  |  |
| Skinkesalat |  |  |  |  |  |  |
| Hamburgerryg |  |  |  |  |  |  |
| Saltkød |  |  |  |  |  |  |
| Spegepølse |  |  |  |  |  |  |
| Kødpølse |  |  |  |  |  |  |
| Teepølse |  |  |  |  |  |  |
| Rullepølse |  |  |  |  |  |  |
| Færdigkøbte frikadeller |  |  |  |  |  |  |
| Kamsteg |  |  |  |  |  |  |
| Leverpostej |  |  |  |  |  |  |
| Paté |  |  |  |  |  |  |
| Kogt medister I skiver |  |  |  |  |  |  |
| Røget svinefilet |  |  |  |  |  |  |
| Røget mørbrad |  |  |  |  |  |  |
| Roastbeef |  |  |  |  |  |  |
| Kylling i skiver |  |  |  |  |  |  |
| Kalkun i skiver |  |  |  |  |  |  |
| Hummus |  |  |  |  |  |  |
| Andet pålæg |  |  |  |  |  |  |

7.5.1 Hvis andet pålæg, hvad__________________________________________________________________________________

**8. FØDEVARER – KØD SPECIALITETER**

8.1 Har du i de **7 dage** før du blev syg spist små snack salamipølser? (Sådan nogle der er lige til at spise og som nogen gange står helt oppe ved disken i supermarkedet og sælges enkeltvis, men de kan også sælges flere i en pakke.)

Ja ☐ Sandsynligvis ja ☐ Sandsynligvis nej ☐	Nej ☐

8.1.1 Hvis ja, Hvilket mærke var det ______________________________________________________________________

8.1.2 hvor var de købt __________________________________________________________________________________

8.1.3 hvordan var pakken (enkelt, flere stk, vaccum) beskriv ____________________________________________________

____________________________________________________________________________________

8.2 Har du i de **7 dage** før du blev syg spist tørret skinke?

Ja ☐ Sandsynligvis ja ☐ Sandsynligvis nej ☐	Nej ☐

8.2.1 Hvis ja, Hvilket mærke var det ______________________________________________________________________

8.2.2 hvor var de købt __________________________________________________________________________________

8.2.3 hvordan var pakken (enkelt, flere stk, vaccum) beskriv ____________________________________________________

____________________________________________________________________________________

8.2.4 hvar det på køl? Ja ☐ Nej ☐

**9. FØDEVARER – ÆG OG MEJERI**

9.1 Har du/din familie i de **7 dage**, inden du blev syg, brugt æg i din husholdning?

Ja ☐ Sandsynligvis ja ☐ Sandsynligvis nej ☐	Nej ☐

9.1.1 Hvis ja eller sandsynligvis ja), var det…

| **Type æg** | **Ja** | **Nej** | **Måske** | **Mærke og beskrivelse af pakke**  (antal, farve på æg….) | **Købt hvor?** |
| --- | --- | --- | --- | --- | --- |
| Buræg |  |  |  |  |  |
| Skrabeæg |  |  |  |  |  |
| Økologiske æg |  |  |  |  |  |
| Æg fra fritgående høns |  |  |  |  |  |
| Pasteuriserede æg |  |  |  |  |  |
| Andet |  |  |  |  |  |

9.1.2 Hvis andet æg, hvad___________________________________________________________________________________

9.2 Har du spist nogle af følgende æggeprodukter i de **7 dage** før du blev syg?

| **Æggeprodukt** | **Ja** | **Sand-synlig-vis ja** | **Sandsynligvis nej** | **Nej** | **Hvis ja eller sandsynligt: Hvilket produkt var det?**  (mærke, producent, økologisk) | **Hvor var æggene købt?** |
| --- | --- | --- | --- | --- | --- | --- |
| Blødkogt æg |  |  |  |  |  |  |
| Hårdkogt æg, fx æggemad |  |  |  |  |  |  |
| Røræg |  |  |  |  |  |  |
| Spejlæg (f.eks på bøf) |  |  |  |  |  |  |
| Porcherede æg |  |  |  |  |  |  |
| Omelet |  |  |  |  |  |  |
| Æggekage |  |  |  |  |  |  |
| Rå æggeblomme |  |  |  |  |  |  |
| Retter med rå æg fx is, koldskål eller mayonaise |  |  |  |  |  |  |
| Retter med pasteuriserede æg |  |  |  |  |  |  |
| Anden æggeret |  |  |  |  |  |  |

9.2.1 Hvis anden æggeret, hvad______________________________________________________________________________________

**10. FØDEVARER – MEJERI**

10.1 Har du spist eller drukket nogle af følgende mejeriprodukter i de **7 dage** før du blev syg?

| **Mejeriprodukt** | **Ja** | **Sand-synlig-vis ja** | **Sandsynligvis nej** | **Nej** | **Hvis ja eller sandsynligt: Hvilket produkt var det?**  (mærke, producent, økologisk) | **Hvor var det købt?** |
| --- | --- | --- | --- | --- | --- | --- |
| Mælk |  |  |  |  |  |  |
| Rå/upastaurise-ret mælk |  |  |  |  |  |  |
| Cacaomælk |  |  |  |  |  |  |
| Crème fraiche |  |  |  |  |  |  |
| Yoghurt |  |  |  |  |  |  |
| Ymer/A38 |  |  |  |  |  |  |
| Is |  |  |  |  |  |  |
| Skæreost |  |  |  |  |  |  |
| Smøreost |  |  |  |  |  |  |
| Rå/upastaurise-ret ost |  |  |  |  |  |  |
| Andet ost |  |  |  |  |  |  |

10.1.1 Hvis andet ost, hvad______________________________________________________________________________________

**11. FØDEVARER – FRUGT OG GRØNT**

11.1 Har du spist nogle af følgende grøntsager i de **7 dage** før du blev syg? Har du spist…

| **Grøntsager** | **Ja** | **Sand-synlig-vis ja** | **Sandsynligvis nej** | **Nej** | **Hvis ja eller sandsynligt: Hvilket produkt var det?** (økologisk, løs vægt, dansk) | **Hvor var det købt?** |
| --- | --- | --- | --- | --- | --- | --- |
| Grøn salat |  |  |  |  |  |  |
| Salatmix |  |  |  |  |  |  |
| Tomat |  |  |  |  |  |  |
| Agurk |  |  |  |  |  |  |
| Rå gulerødder |  |  |  |  |  |  |
| Færdigpakket, klar-til-at-spise salat |  |  |  |  |  |  |
| Færdigpakket, klar-til-at-spise andre rå grøntsager |  |  |  |  |  |  |
| Færdigpakket, klar-til-at-spise pastasalat el kartoffelsalat |  |  |  |  |  |  |
| Andre grøntsager |  |  |  |  |  |  |

11.1.1 Hvis andre grøntsager, hvad _______________________________________________________________________________

11.2 Har du spist nogle af følgende frugter i de **7 dage** før du blev syg? Har du spist…

| **Frugt** | **Ja** | **Sand-synlig-vis ja** | **Sandsynligvis nej** | **Nej** | **Hvis ja eller sandsynligt: Hvilket produkt var det?** (slags, økologisk, løs vægt, dansk) | **Hvor var det købt?** |
| --- | --- | --- | --- | --- | --- | --- |
| Melon |  |  |  |  |  |  |
| Æbler |  |  |  |  |  |  |
| Jordbær |  |  |  |  |  |  |
| Hindbær |  |  |  |  |  |  |
| Frosne bær |  |  |  |  |  |  |
| Færdigpakket, klar-til-at-spise frugtsalat |  |  |  |  |  |  |
| Fædigpakket, klar-til-at-spise andet rå frugt |  |  |  |  |  |  |
| Frugtjuice |  |  |  |  |  |  |
| Smoothies |  |  |  |  |  |  |
| Andet frugt |  |  |  |  |  |  |

11.2.1 Hvis andet frugt, hvad___________________________________________________________________________________

**12. FØDEVARER KRYDDERURTER & KRYDDERIER**

12.1 Har du spist nogle af følgende krydderurter i de **7 dage** før du blev syg? Har du spist…

| **Frugt** | **Ja** | **Sand-synlig-vis ja** | **Sandsynligvis nej** | **Nej** | **Hvis ja eller sandsynligt: Hvilket produkt var det?** (slags, økologisk, løs vægt, dansk) | **Hvor var det købt?** |
| --- | --- | --- | --- | --- | --- | --- |
| Frisk basilikum |  |  |  |  |  |  |
| Persille |  |  |  |  |  |  |
| Koriander |  |  |  |  |  |  |
| Purløg |  |  |  |  |  |  |
| Rosmarin |  |  |  |  |  |  |
| Timian |  |  |  |  |  |  |
| Spirer fx bønnespirer |  |  |  |  |  |  |
| Karse |  |  |  |  |  |  |
| Anden krydderurt |  |  |  |  |  |  |

12.1.1 Hvis andet krydderurt, hvad ________________________________________________________________________________

12.2 Har du spist nogle af følgende tørrede krydderier i de **7 dage** før du blev syg? Har du spist…

| **Krydderi** | **Ja** | **Sand-synlig-vis ja** | **Sandsynligvis nej** | **Nej** | **Hvis ja eller sandsynligt: Hvilket produkt var det?** (økologisk, løs vægt, dansk) | **Hvor var det købt?** |
| --- | --- | --- | --- | --- | --- | --- |
| Karry |  |  |  |  |  |  |
| Paprika |  |  |  |  |  |  |
| Spidskommen |  |  |  |  |  |  |
| Oregano |  |  |  |  |  |  |
| Anis |  |  |  |  |  |  |
| Muskatnød |  |  |  |  |  |  |
| Sesamfrø |  |  |  |  |  |  |
| Tahin |  |  |  |  |  |  |
| Andre krydderier |  |  |  |  |  |  |

12.2.1 Hvis andre krydderier, hvad_________________________________________________________________________________

**13. FØDEVARER – SLIK OG SNACKS**

13.1 Har du spist nogle af følgende typer slik og nødder i de **7 dage** før du blev syg? Har du spist…

| **Snacks/slik** | **Ja** | **Sand-synlig-vis ja** | **Sandsynligvis nej** | **Nej** | **Hvis ja eller sandsynligt: Hvilket produkt var det?** (mærke, lys/mørk, hvordan så posen/pakken ud?) | **Hvor var det købt?** |
| --- | --- | --- | --- | --- | --- | --- |
| Chokolade |  |  |  |  |  |  |
| Pålægschokolade |  |  |  |  |  |  |
| Chokolade bar |  |  |  |  |  |  |
| Peanutbuter |  |  |  |  |  |  |
| Protein- eller müslibar |  |  |  |  |  |  |
| Hasselnødder |  |  |  |  |  |  |
| Valnødder |  |  |  |  |  |  |
| Mandler |  |  |  |  |  |  |
| Pinjekerner |  |  |  |  |  |  |
| Andre nødder fx saltede |  |  |  |  |  |  |
| Chips |  |  |  |  |  |  |
| Andet slik |  |  |  |  |  |  |

13.1.1 Hvis andet slik, hvad___________________________________________________________________________________

**14. DYR**

14.1 Har du/familien nogle husdyr, kæledyr eller krybdyr? Ja ☐ Nej ☐

14.1.1 Hvis ja, hvilke _________________________________________________________________________________________

14.1.2 Hvad får dyret at spise? _________________________________________________________________________________

14.2 Har du i øvrigt haft kontakt (rørt ved) med nogle dyr inkl. krybdyr i de **7 dage** før du blev syg?

Ja ☐ Sandsynligvis ja ☐ Sandsynligvis nej ☐	Nej ☐

14.2.1 Hvis ja, hvilke dyr? _______________________________________________________________________________________

**16. KONTAKT**

Så har jeg ikke flere spørgsmål. Til aller sidst vil jeg blot spørge dig om:

16.1 Må vi kontakte dig igen på et senere tidspunkt, hvis vi har flere spørgsmål? Ja ☐	Nej ☐

**Du skal have mange tak for hjælpen, fortsat god dag/aften**
